# Trackway evidence for large bipedal crocodylomorphs from the Cretaceous of Korea

**DOI:** 10.1038/s41598-020-66008-7

**Published:** 2020-06-11

**Authors:** Kyung Soo Kim, Martin G. Lockley, Jong Deock Lim, Seul Mi Bae, Anthony Romilio

**Affiliations:** 1 Department of Science Education, Chinju National University of Education, Shinan-dong, Jinju, Kyungnam 52673 South Korea; 2 Dinosaur Trackers Research Group, University of Colorado Denver, P.O. Box 173364, Denver, CO 80217-3364 USA; 3 Restoration Technology Division, National Research Institute of Cultural Heritage, 132, Munji-ro Yuseong-gu, Daejon, 34122 South Korea; 4 Institute of Korea Geoheritage, Chinju National University of Education, Shinan-dong, Jinju, Kyungnam 52673 South Korea; 5 School of Chemistry and Molecular Biosciences, The University of Queensland, Brisbane, Qld 4072 Australia

**Keywords:** Palaeontology, Sedimentology

## Abstract

Large well-preserved crocodylomorph tracks from the Lower Cretaceous (? Aptian) Jinju Formation of South Korea, represent the well-known crocodylomorph ichnogenus *Batrachopus*. The Korean sample includes multiple, narrow-gauge, pes-only trackways with footprint lengths (FL) 18–24 cm, indicating trackmaker body lengths up to ~3.0 m. Surprisingly, the consistent absence of manus tracks in trackways, with well-preserved digital pad and skin traces, argues for bipedal trackmakers, here assigned to *Batrachopus grandis* ichnosp. nov. No definitive evidence, either from pes-on-manus overprinting or poor track preservation, suggests the trackways where made by quadrupeds that only appear bipedal. This interpretation helps solve previous confusion over interpretation of enigmatic tracks of bipeds from younger (? Albian) Haman Formation sites by showing they are not pterosaurian as previously inferred. Rather, they support the strong consensus that pterosaurs were obligate quadrupeds, not bipeds. Lower Jurassic *Batrachopus* with foot lengths (FL) in the 2–8 cm range, and Cretaceous *Crocodylopodus* (FL up to ~9.0 cm) known only from Korea and Spain registered narrow gauge trackways indicating semi-terrestrial/terrestrial quadrupedal gaits. Both ichnogenera, from ichnofamily Batrachopodidae, have been attributed to *Protosuchus*-like semi-terrestrial crocodylomorphs. The occurrence of bipedal *B. grandis* ichnosp. nov. is evidence of such adaptations in the Korean Cretaceous.

## Introduction

Crocodylomorph tracks are generally rare in the Mesozoic of Asia. It has been suggested that this is in part due to the lack of sedimentary facies representing suitable habitats for this group of ostensibly aquatic trackmakers^1^. However, the crocodylomorph ichnofamily Batrachopodidae^2,3^ appears to represent more terrestrially-adapted forms^3,4^. As currently defined, the batrachopodids^5^ include *Batrachopus* and *Crocodylopodus*, the former mostly known from small tracks (~2.0–8.0 cm long) from the Lower Jurassic of North America^4^, Europe^5,6^ and Africa^7^, the latter primarily from the Cretaceous of Europe^8,9^. The ichnofamily also includes *Antipus* known only from one well-described Lower Jurassic trackway from North America which is considered a synonym of *Batrachopus* by some^3^, but not all ichnologists^10^.

Recently *Crocodylopodus* was reported from the Lower Cretaceous (?Aptian)^11^ Jinju Formation of Korea^12,13^, where it represents the first Asian occurrence, and adds to the extraordinarily rich Jinju Formation, ichnofauna described as a Konservat-Lagerstätten^14–19^. Spanish^8,9^ and Korean^12,13^ *Crocodylopodus* represent relatively small animals (footprint lengths less than ~9.0–10.0 cm) with slender digit traces, which may reflect penetrative track preservation^20^. However, the trackway configuration is diagnostically crocodilian, with elongate tetradactyl pes and outwardly rotated pentadactyl manus, not unlike those of extant crocodilians^21–23^ (Supplementary Information: SI Fig. 1).

**Figure 1:**
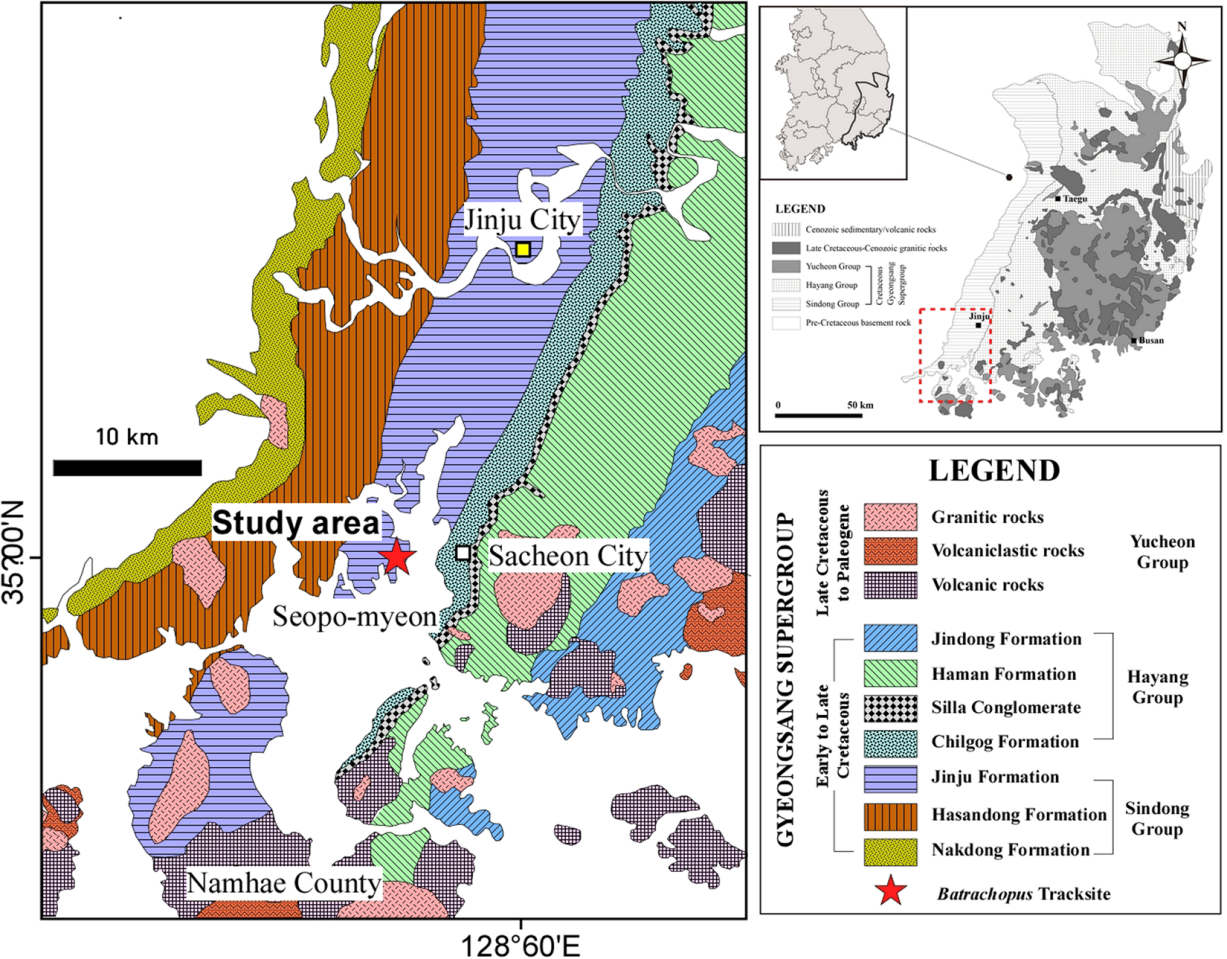
Location maps and stratigraphy for the Gyeongsang Supergroup. (**A**) location of the Gyeongsang Basin in southeast of Korean Peninsula, with Group-level geological map. (**B**) Formation-level geological map of the southwestern part of the Gyeongsang Basin showing study area (Sacheon Jahye-ri tracksite) west of Sacheon City. (**C**) Formation-level stratigraphy of region. Maps made by K-S K and M G L in Adobe Photoshop (version CS6 88) and Canvas X (version, 20 Build 390, http://www.canvasgfx.com/).

Here we report on a large, newly-discovered Jinju Formation assemblage with multiple trackways of large crocodylomorph tracks with footprint lengths up to 24.0 cm, from the Sacheon Jahye-ri tracksite, near Sacheon City (Fig. 1). These tracks are more than twice as large as any previously reported batrachopodid tracks and closely resemble *Batrachopus* with well-preserved pes footprints with clear digital pad impressions and localized skin traces. Surprisingly the trackways never include manus imprints and therefore appear to indicate exclusively bipedal progression, a gait not known or previously inferred from fossil crocodylomorph trackways, or argued convincingly from the functional morphology of potential trackmakers (SI).

In contrast to the common Mesozoic crocodylomorph trackway *Hatcherichnus*^24^, representing swimming behavior, and it’s rare walking counterpart *Mehliella*^25^, *Batrachopus* has been attributed to a *Protosuchus*-like crocodylomorph^3,4^ with a narrow trackway interpreted to represent terrestrial progression. Evidence of bipedalism is consistent with such a terrestrial gait.

The unexpected discovery of trackways so suggestive of bipedal locomotion by Cretaceous crocodylomorphs has ichnological implications bearing directly on long-standing and controversial debates about the gait of pterosaurs. While, most pterosaurian trackways represent quadrupedal^26^ not bipedal progression^27^, multiple pes-only trackways, from the Haman Formation of Korea have been erroneously attributed to giant bipedal pterosaurs^28^. This unexpected evidence of apparently bipedal crocodylomorphs obliges us to investigate the alternative possibility that the trackway configurations represent an unusual mode of preservation, rather than bipedal locomotion, and underscores the need for a reexamination of the Batrachopodidae^29^ and other relevant, morphologically-similar ichnotaxa.

## Ichnological material and geological setting

**Figure 2:**
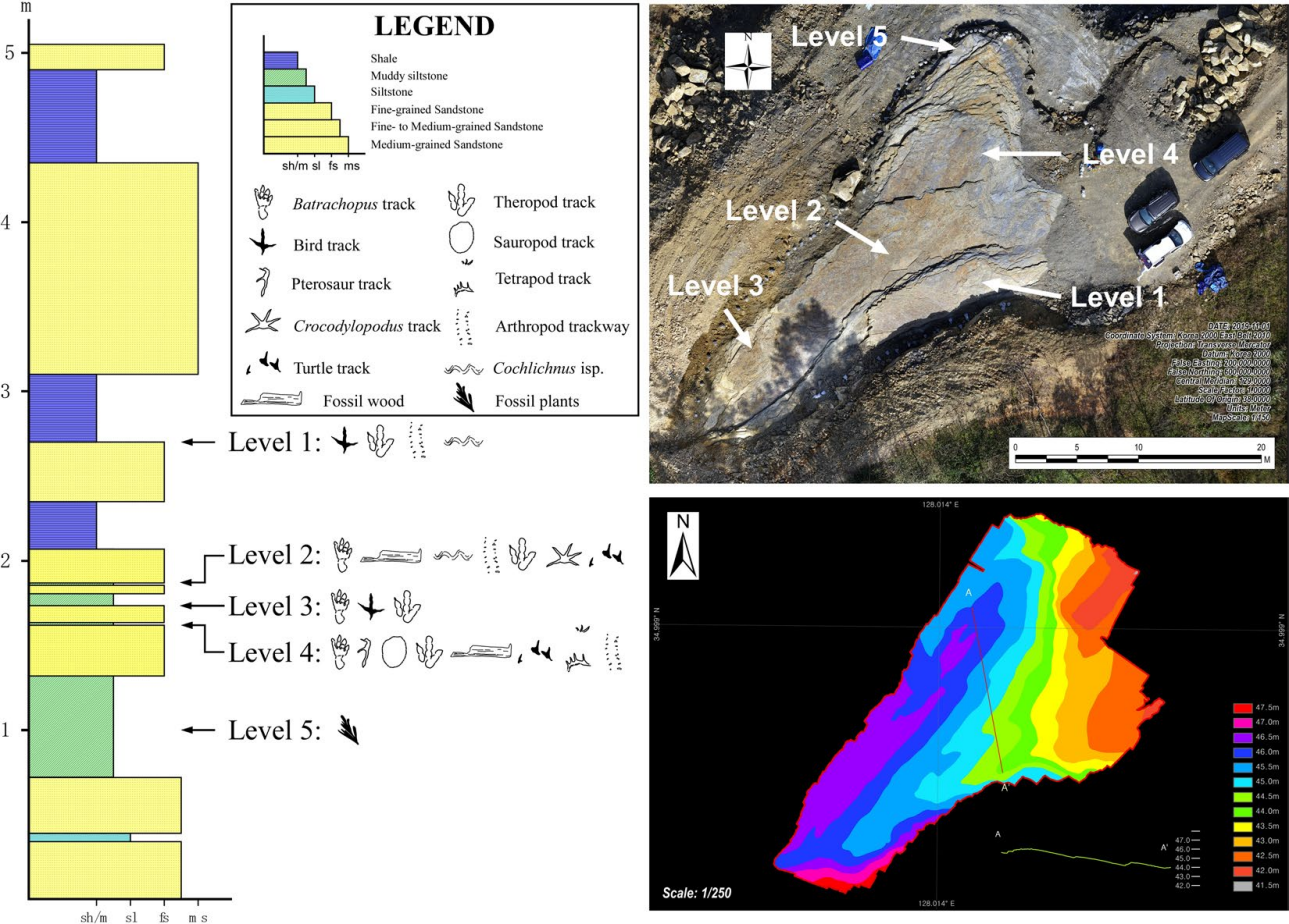
Stratigraphy (left) of the Sacheon Jahye-ri tracksite with aerial view (top right), with elevation of surface (bottom right) showing cross section with main stratigraphic layers. Graphics made by K-S K and M G L in Adobe Photoshop (version CS6 88) and Canvas X (version, 20 Build 390, http://www.canvasgfx.com/). Photographs for the aerial view collected with a Sony A7R digital camera (25 mm lens) and the elevation images of surface using Agisoft PhotoScan Professional (v. 1.4.3) and esri ArcMap (v. 10.2.2.).

In recent years the Lower Cretaceous (?Aptian) Jinju Formation, in the Jinju City area of South Korea has yielded an extraordinary volume and diversity of tetrapod tracks including “first” discoveries of small hopping mammal tracks, *Koreasaltipes jinjuensis*^14^, truly diminutive dromaeosaur tracks *Dromaeosauriformipes rarus*^15^, the first Korean examples of the theropod track *Corpulentapus*^16^, the first turtle tracks from Korea^30^, the aforementioned first report of *Crocodylopodus*^12^, the oldest known frog tracks^18^, diminutive theropod tracks (*Minisauripus*) with skin traces^19^, and the largest known Cretaceous lizard track assemblage^17^. While many other track assemblages and facies remain under active investigation, the present study focusses on the documentation of the large batrachopodid tracks and trackways from the Sacheon Jahye-ri tracksite (Figs. 1 and 2) from which large numbers of large *Batrachopus* tracks were recovered. This is the first global report of large *Batrachopus* and the first to indicate bipedal progression. They form the basis of the new ichnotaxon *Batrachopus grandis* ichnosp. nov., described in detail below.

**Figure 3:**
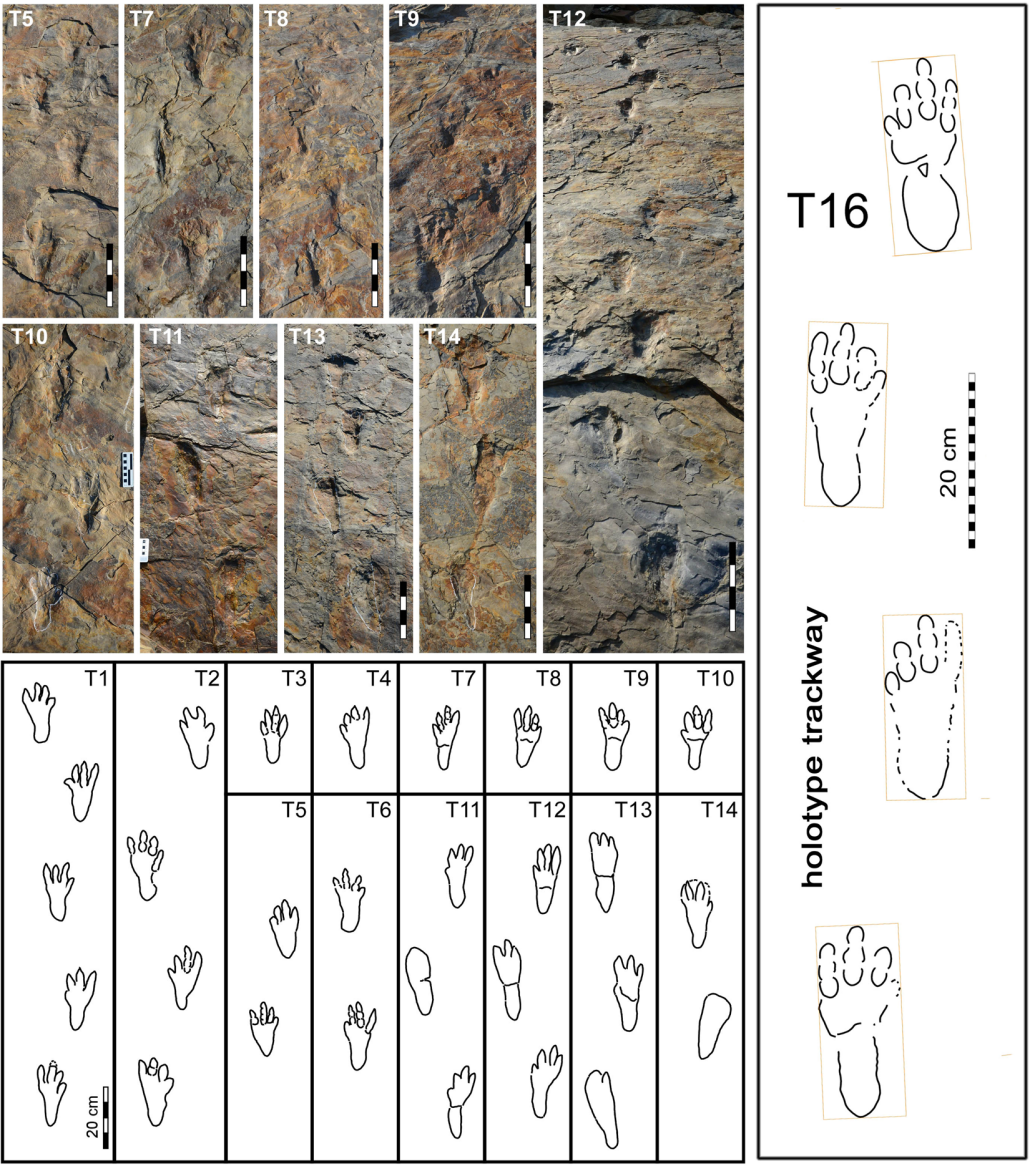
Photos and outline drawings of trackways and trackway segments of *Batrachopus grandis* ichnosp. nov. from *in situ* outcrops. T1-T10 and holotype trackway T16 (enlarged, with length and width rectangle indicating minimal rotation) represent level 2 and T11-T14 represents level 4. Compare with Figs. 4–6, SI Figs. 3 and 4 and SI Table 1. Photos and line drawings made and compiled by K-S K and M G L, in Adobe Photoshop (version CS6 88) and Canvas X (version, 20 Build 390, http://www.canvasgfx.com/).

**Figure 4:**
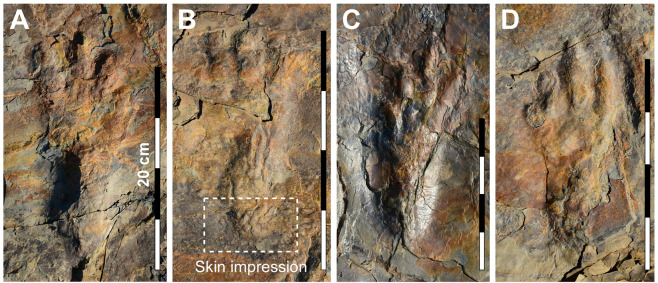
Photographs of well-preserved *in situ Batrachopus grandis* ichnosp. nov. track impressions from surface 2. (**A**,**B**) Left pes tracks; (**C**,**D**) Right pes tracks. A, C = pes of T7 and T9, respectively. B shows skin impression of heel region (see SI Fig. 5). D = holotype IS 2 of Fig. 6. Photos by J-W K, S-M B and M G L, and compiled in Adobe photoshop (version CS6 88) and Canvas X (version, 20 Build 390, http://www.canvasgfx.com/).

**Figure 5:**
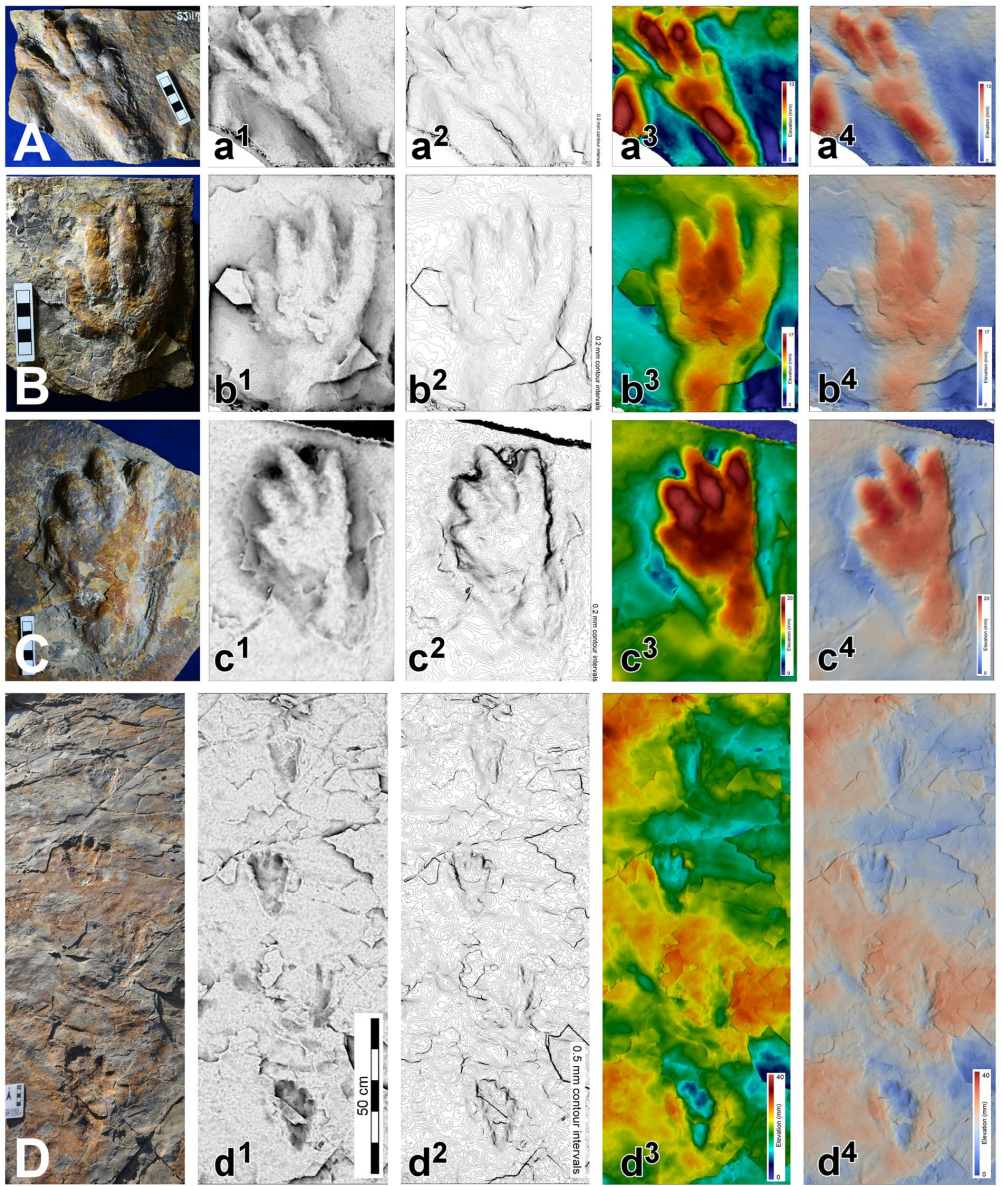
Photos and 3D images of track casts and trackway segments. (**A–D**): Photos of SJ 117, 119, 138 and trackway 1; (a–d): 3D images of SJ 117, 119, 138 and trackway 1. Compare with Fig. 3 and SI Figs. 3 and 4. Photos and 3D images made and compiled by K-S K, S-M B and A R, in Adobe photoshop (version CS6 88) and Canvas X (version, 20 Build 390, http://www.canvasgfx.com/).

**Figure 6:**
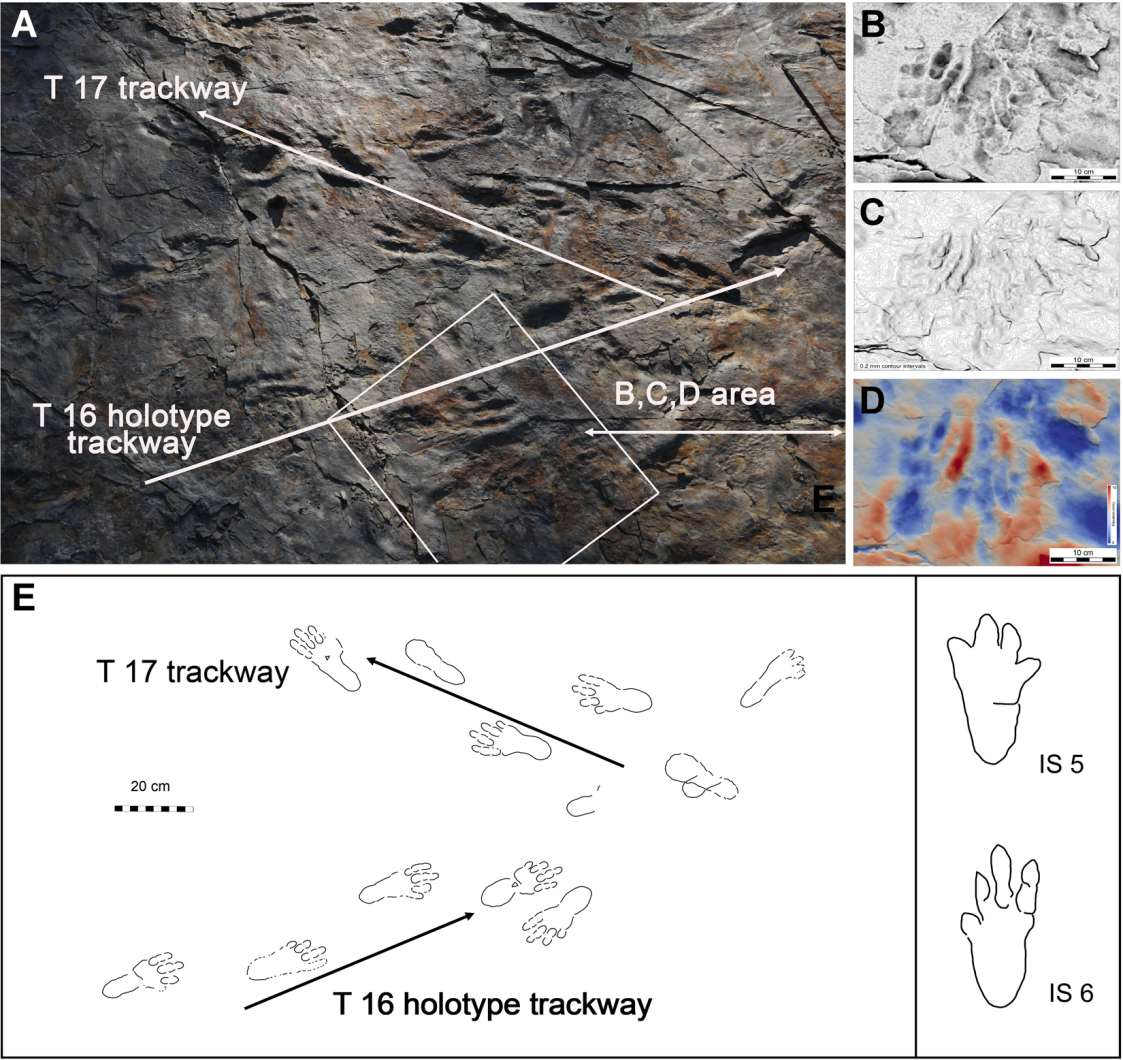
(**A**) Photograph of the surface from which tracings and 3D images of trackways T16 (the holotype) and T17 of *Batrachopus grandis* and isolated tracks IS 5-6 were obtained; (**B**–**D**) 3D images of pair of tracks including right pes track from holotype trackways T 16; (**E**) map of the surface shown in A, showing holotype trackway T 16 and paratype trackway T 17. Note well-defined digital pad traces especially in holotype. Photographs, line drawings and 3D images made and compiled by K-S K, M G L and A R. in Adobe Photoshop (version CS6 88) and Canvas X (version, 20 Build 390, http://www.canvasgfx.com/). Compare with Figs. 3–5, SI Figs. 3 and 4 and SI Table 1.

The Sacheon Jahye-ri tracksite represents an area of excavation approximately 40 × 20 meters (800 m^2^) in extent which has yielded four track-bearing levels from a outcrop and representing about 5 meters of stratigraphic section within the Jinju Formation (Fig. 2). Tracks are abundant at all levels, with high concentrations of crocodylomorph (*Batrachopus*) tracks and trackways at levels 2 and 4 (Figs. 3–6).

Standard methods of *in situ*, and laboratory track and trackway documentation, were used to document the large volume of ichnological material collected, the largest *Batrachopus* sample currently known (SI). The track-bearing layers were necessarily exposed by working from higher to lower stratigraphic levels, and resulted in removal of a large number of natural casts (convex hyporeliefs) that had filled the natural impressions (concave epireliefs) on successively lower surfaces including levels 2 and 4 from which many specimens, mostly casts were collected. Comprehensive methods of documentation and analysis described in the Supplementary Information, included 2D photography, 3D photogrammetry, tracing of track outlines and measurement of all standard track and trackway parameters (Figs. 3–6 and SI Figs. 3 and 4, SI Table 1).

### Description of tracks and trackways

#### General observations

The tracks here formally referred to *Batrachopus grandis* ichnosp. nov., are all pes traces, occurring in narrow trackways (Fig. 3) and are consistently about twice as long as wide (Figs. 4–6) when including the heel trace which appears to be registered in all cases. Given the natural morphological division of the pes tracks into an anterior and posterior portion separated by a transverse crease (Figs. 4–6), it is possible to provide length and width measurements for both the entire track, including the heel and the anterior portion (SI Table 1). Providing both measurements is important in making comparisons with Jurassic *Batrachopus*, including the type material from North America, which only reveals sporadically or faintly preserved heel traces. In some cases the heel trace is absent and the distinctive bi-lobed posterior margin of the anterior portion of the tracks is clearly seen. In the following description, the lengths of digit traces and their inter-digital divarication angles are all measured from the posterior-most margin of the heel (SI Fig. 2).

Field observations indicated some differences in the quality of preservation of *B. grandis* ichnosp. nov. tracks from the level 2 and 4 surfaces. General tracks from the level 2 surfaces were shallower with clearly defined digit and digital pad impressions (Figs. 3–6), as well as transverse creases. They also yielded the majority of well-preserved natural casts. The well-defined digit and pad traces, rank these tracks as 2.5–3.0 on the four point preservation scale (0-1-2-3)^31,32^. One track shows well-defined skin (scale) impressions in the heel area that resemble those typical of modern crocodilians (Fig. 4B and SI Fig. 5)^21–23^. The tracks from level 4 were generally deeper and slightly more elongate, which we infer to be related to softer substrate conditions: see trackways T11–T14 (Fig. 3). In these trackways, which are slightly narrower, with corresponding higher pace angulation values, digit traces are still quite well-preserved, although digital pad traces are less well-defined.

### Systematic description

Batrachopodidae Lull 1904, emended Lockley and Meyer 2004.

*Batrachopus grandis* ichnosp. nov.

Holotype: Trackway T 16 (Figs. 3, 4D and 6A. Table 1); with two replicas, CUE SJ IS 2 R and UCM 214.326

**Table 1:** Occurrence of named trackways: *Antipus*, *Batrachopus*, *Crocodylopodus, Hatcherichnus, Kuangyuanpus* and *Mehliella* of purported crocodylomorph affinity from the Mesozoic of North America, Europe and Asia.

Track ichnotaxonomy	Rock unit and Locality	Age	Swimming (S) or walking (W) trace	Reference
Albertasuchipes*	Paskapoo Fm., Canada	Paleocene	**S**	^57^
Anticusuchipes*	Chuckanut Fm., Washington	Eocene	**S**	^48^
Borealosuchipus*	Bullion Creek Fm., N. Dakota	Late Paleocene	**S**	^58^
Indosuchipes*	Laisong Fm., India	Eocene - Oligocene	**S**	^59^
unnamed	Lance Fm., Wyoming	Upper Cretaceous	**S**	^60^
*Hatcherichnus*	Blackhawk Fm., Utah	Upper Cretaceous	**S**	^61^
cf. *Crocodylopodus*	Wahweap Fm., USA	Upper Cretaceous	?	^62^
?*Hatcherichnus*	Dunvegan Fm., Canada	Upper Cretaceous	**S**	^63^
Mehliella*	Dakota Gp., Colorado	‘mid’ Cretaceous	W	^25,64^
*Hatcherichnus*	Dakota Gp., Colorado	‘mid’ Cretaceous	**S**	^49^
***Batrachopus grandis*** **ichnosp. nov**.	**Jinju Fm., S. Korea**	**Lower Cretaceous**	W	**This paper**
*Crocodylopodus*	Jinju Fm., S. Korea	Lower Cretaceous	W	^12,13^
?*Hatcherichnus*	Gates Fm., Canada	Lower Cretaceous	S	^63^
?*Hatcherichnus*	Mist Mtn. Fm., Canada	Lower Cretaceous	S	^63^
*Batrachopus*	Thailand	Lower Cretaceous	W	^29^
?*Crocodylopodus*	Huerteles, Spain	Lower Cretaceous	W	^9^
Crocodylopodus*	Spain	Lower Cretaceous	W	^8^
Hatcherichnus*	Morrison Fm., Utah, USA	Upper Jurassic	S	^24^
*Crocodylopodus*	Lastres Fm., Spain	Upper Jurassic	W	^65^
*Crocodylopodus*	Hojedk Fm. Iran	Upper Jurassic	W	^66^
Kuangyuanpus*	Shandong, China	Middle Jurassic	? W	^67^
*Batrachopus*	Glen Cyn. Gp. USA	Lower Jurassic	W	^68^
Antipus*	Eastern USA	Lower Jurassic	W	^10^
Batrachopus*	Eastern USA	Lower Jurassic	W	^3^

The other named crocodylomorph trackways are *Albertasuchipes*, *Anticusuchipes*, *Borealosuchipus* and *Indosuchipes* from Paleocene through Oligocene. *Indicates type material. Table is arranged in descending stratigraphic order, and differentiates swimming (S) and walking (W) traces.

Paratypes: Trackways T1-T14 and T17 (Fig. 3, Table 1) and isolated (IS) tracks in Sacheon Jahye-ri (SJ) series SJ 001 – SJ 140 (SI Fig. 3).

Type locality: the Sacheon Jahye-ri tracksite, near Sacheon City, South Korea

Type horizon: Jinju Formation, Lower Cretaceous (?Aptian)^11^.

### Differential diagnosis

*B. grandis* ichnosp. nov. is much larger than all previously described *Batrachopus* ichnospecies and morphotypes, mostly ‘lumped’ under the Lower Jurassic ichnogenus *B. deweyi*^3^, and differs from them in consistently displaying a well-defined heel making up on average ~40% of total pes length. *B. grandis* ichnosp. nov. is also less outwardly rotated than type *Batrachopus* (*B. deweyi*): i.e., in the former morphotype digit III parallels the trackway mid line, not digit II as in the latter ichnospecies with more outwardly rotated pes. No other *Batrachopus* ichnospecies has been described in such detail, from such a large and well preserved sample, even with skin traces, as Korean *B. grandis* ichnosp. nov. This allows for the definition of anatomy-consistent and diagnostic features such as the relative heel length and relative digit divarication angles with III-IV consistently greater than I-II and II-III (SI Fig. 3): i.e., ∠III-IV > ∠I-II and ∠II-III.

### Description

Trackway of a tetrapod with tetradactyl pes. The manus traces were not found but register as pentadactyl traces in other *Batrachopus* ichnospecies. Pes twice as long as wide with narrow heel, separated from wider anterior portion, with digit traces, by a transverse, bi-lobed crease with anteriorly convex sulcus. Total track length averaging 20.7 cm (range 18.0–24.0 cm), along axis of longest digit III, with mean lengths of digits I, II and IV respectively as 14.7, 18.6 and 19.4 cm (from posterior margin of heel). Thus, III > IV > II > I. Digit traces up to ~2.0 cm wide, with well-defined pad impressions, generally in the better preserved specimens indicating a phalangeal pad formula of 1-2-3-4 corresponding to digits I, II, III and IV. However, the creases that differentiate the pad traces are not always clearly registered, especially in digit IV. Length of that portion of the track anterior to the transverse crease averaging 12.2 cm (range 11.0–15.1 cm): thus L/W ratio of anterior digital portion of the pes is 1.27 (12.2 cm/9.6 cm), with maximum track width situated anteriorly near distal margins of digits I and IV. Total I-IV digit divarication averaging 31.2° (range 28.0–38.0°) with mean divarication values for I-II, II-III and III-IV as 9.3°, 9.4° and 12.3° respectively: (∠III-IV > ∠I-II and ∠II-III). Skin traces consist of angular polygonal scale traces about 1.0 cm in diameter, and are presently known in the heel region.

Trackway narrow without manus traces on surfaces where pes traces registered. Step averaging 37.3 cm (range 29.8–47.0 cm); stride averaging 69.3 cm (range 50.8–81.0 cm); pace angulation averaging 148.5° (range 135–165°); outer trackway width (OTW) averaging 18.7 cm and inner trackway width averaging 0.6 cm and ranging from 5.5 to - 4.0 cm (SI Table 1).

#### Systematic discussion

Heel traces occur sporadically in type *Batrachopus*, i.e., *B. deweyi* from the Lower Jurassic of the Connecticut Valley region^3^, and are regarded as possibly incorporating a reduced digit V, never illustrated as a separate digit trace^3,33^. However they are inconsistently and incompletely registered, thus appearing in only two of seven consecutive tracks in the type trackway, and never with skin traces. In the case of the type of *B. “gracilis”* trackway^3^ there are no heel traces in this trackway, as is the case for most other known examples^5,6,26^.

*Crocodylopodus* described from the basal Cretaceous of Spain^8^ is based on a well-defined holotype trackway with tetradactyl pes, only 3.6 cm long with narrow digit traces that do not show pad impressions or any skin traces (SI Fig. 1B). The manus is pentadactyl and outwardly rotated, also with very narrow digit traces. Ostensibly the narrow digit traces distinguish *Crocodylopodus* from *Batrachopus*, with the former showing less outward rotation of the pes, but greater outward rotation of the manus tracks. If the difference in digit trace width is explained as a preservation feature, with the narrow traces being considered penetrative tracks^20^, the difference between *Batrachopus* and *Crocodylopodus* may be explained in part by differential preservation.

Recent studies of crocodylomorph tracks from the Cretaceous of Korea, notably from other Jinju Formation sites, have applied the term *Crocodylopodus* to small tracks (pes length less than 9.0 cm) with narrow digit traces that closely resemble Spanish *Crocodylopodus*^12,13^ (SI Fig. 6). These Korean *Crocodylopodus* trackways represent quadrupedal progression and none represent animals even half the size, in linear footprint dimensions, of the *B. grandis* ichnosp. nov. trackmaker. Although both the large and the small Korean crocodylomorphs tracks are considered batrachopodids, there is, as yet, no compelling case that their ichnogenus or ichnospecies level taxonomies are indistinguishable, or that the trackmakers (of *B. grandis* ichnosp. nov. and Korean *Crocodylopodus*) were the same. In order to make a case for strong similarity, cogent arguments for the role of preservational factors are needed, and discussed below.

## Discussion

### Evidence for bipedalism of the *B. grandis* ichnosp. nov. trackmaker

Various crocodylomorph tracks have been reported from the Mesozoic and Cenozoic^13,22^ and assigned to at least eleven ichnogenera (Table 1) of which six occur exclusively in the Jurassic and Cretaceous. The discovery of *B. grandis* ichnosp. nov. was made soon after the discovery and description of Korean *Crocodylopodus*^12,13^ and significantly enhances our understanding of the morphology, size range, abundance and preservational factors affecting Korean crocodylomorph tracks: i.e., Batrachopodidae footprints (Table 1), and more generally to the range of morphotypes attributable to this ichnofamily.

From an ichnotaxonomic viewpoint it has already been argued that *B. grandis* ichnosp. nov. can be differentiated from all other reports of *Batrachopus*. It differs from other morphotypes not only in size, but in being more plantigrade: i.e., always registering heel traces both in shallower and deeper tracks and in showing less outward rotation of the pes (Fig. 6). It also has very well-preserved digital pad traces and even some scale impressions. If the trackmaker was truly bipedal (either an obligate or facultative biped) as seems persuasive based on the consistent biped configuration in trackways, a case could be made for a higher level of taxonomic differentiation, say at the ichnogenus level: i.e. bipedalism would be another diagnostic difference. We first examine the possibility of bipedality and, second, consider the alternate possibilities.

**Figure 7:**
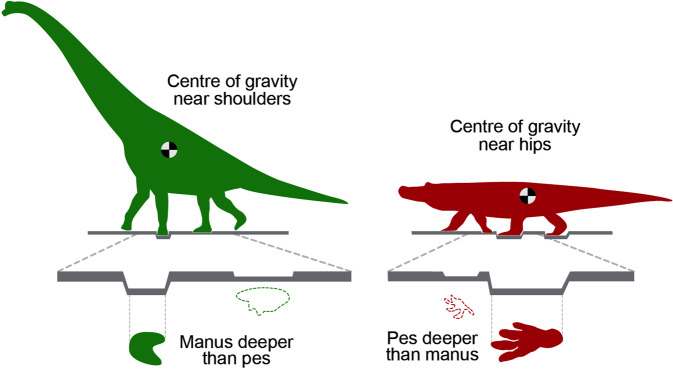
How differential depth of manus and pes may affect trackway configurations. Note manus only and manus dominated trackways are common in the sauropod track record (left). The possibility of a pes deeper than manus scenario to explain apparent evidence of a bipedal crocodilian (right) is considered a possibly, but highly unlikely. See text for details. Original graphics made and compiled by M G L and A R, in Adobe Photoshop (version CS6 88) and Canvas X (version, 2017 Build 160, http://www.canvasgfx.com/).

It is well known that in addition to obligate bipeds and obligate quadrupeds, there are also various facultative bipeds and quadrupeds. Among the archosaurs we recognized that some sauropodomorphs (“prosauropods”) were facultative bipeds, as shown by the occurrence of both pes-only and manus-and-pes trackways of *Otozoum*^34,35^. It is also well known that various ornithischians were facultative quadrupeds, as demonstrated by the various examples of trackways assigned to the ichnogenera *Anomoepus*, *Moyenisauropus*^36^ and *Caririchnium* indicate^37^. In all these cases the manus tracks are much smaller than those of the pes, which appears to have been universally the case with terrestrial archosaurs. This is true not only of those described as “small manus” forms with greater heteropody, but also among “large manus” morphotypes with less heteropody^38–41^. From this it is possible to infer that most forms carried most of their weight and over the pelvic girdle, and it has been noted that an elongate heel trace is often associated with bipedal forms: i.e., as an expression of posture with posterior emphasis^41^. However, as discussed for sauropods^41^ the force exerted on the substrate by manus and pes is a function of both weight and foot size: i.e., weight distribution. Thus, in the case of sauropods, it was shown, both in theory and in the rock record that the manus could penetrate more deeply that the pes (Fig. 7), creating different impacts on different sedimentary layers, in some cases creating “manus only” trackways^42^. This largely dispelled speculations that manus-only trackways were the result of sauropod swimming activity. Other, compelling lines of evidence suggesting that the Korean *B. grandis* trackways are attributable to bipedal crocodylomorphs come from the known occurrence of large bipedal crocodylomorphs from the early Mesozoic^43,44^. However, to date no such bipedal crocodylomorphs have been reported from the Cretaceous.

### Possible arguments against bipedalism of the *B. grandis* trackmaker

Although the sauropod swim tracks debate need not be reviewed in detail here (Fig. 7), it is pertinent to note that the pes-only *B. grandis* ichnosp. nov. trackways can only logically be attributed to deeper penetration of the pes. If the manus had penetrated more deeply, or as deeply, as in the case of some sauropods, one would expect to see deeper manus traces on the same surfaces as the pes tracks. There is no evidence for this at the Sacheon Jahye-ri tracksite.

This argument leads logically to only a few possible conclusions. The first is that the manus did register footprints as the trackmaker progressed quadrupedally, but that *the manus did not penetrate as deeply as the pes*, because it exerted less force: i.e., weight was distributed mostly over the pes, beneath the pelvic, rather than the pectoral girdle (Fig. 7). This inference, which implies that the manus registered on stratigraphically higher surfaces, which perhaps had different compositions, consistencies and preservation potential, is potentially consistent with the evidence that all other batrachopodid trackways reveal a smaller manus than pes. But such a scenario assumes that the large track sinks deeper, and does not factor in size and weight distribution differences. There is no documented evidence to suggest that the manus was less clearly or deeply impressed than the pes in small quadrupedal batrachopodid trackways. So there is no direct evidence that the pes tracks penetrated to register on surfaces buried beneath the surfaces on which the manus tracks are inferred to have registered. Therefore, this interpretation, implying that all manus tracks registered on surfaces other than those on which the pes is consistently found, is also very difficult to support in the light of the recognition of other tracksites, discussed below, with similar evidence of similar ostensibly bipedal large batrachopodids.

**Figure 8:**
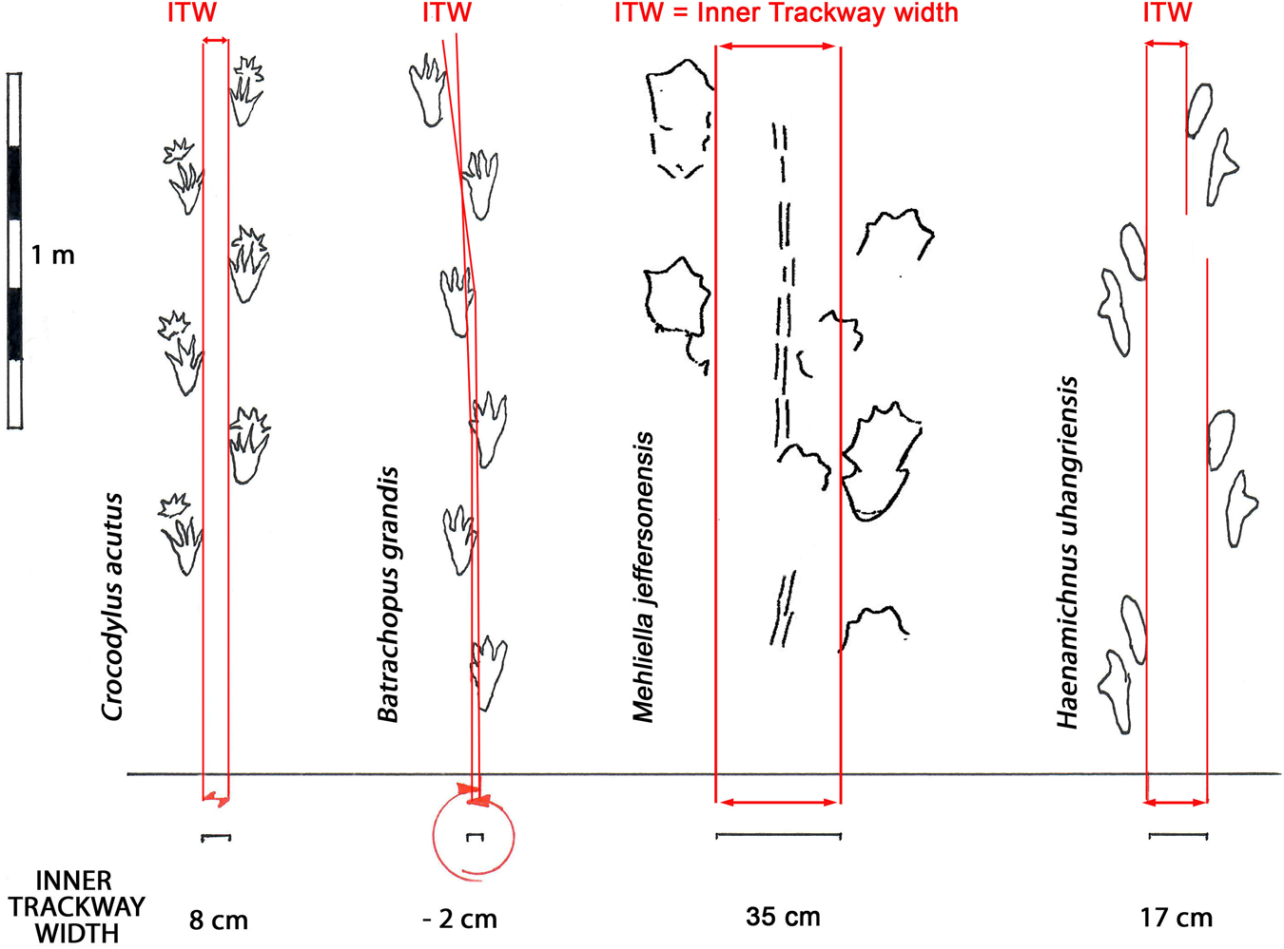
Left to right: Comparison of trackways of modern *Crocodylus acutus*^45^*, Batrachopus grandis* ichnosp. nov., *Mehliella jeffersonensis* (with tail trace)^25^ and the pterosaur trackway *Haenamichnus uhangriensis* (right)^56^, showing difference in trackway width. Note that *B. grandis* ichnosp. nov. trackway indicates a biped and that the trackway is narrow with inner trackway width negative. Original line drawings made and compiled by M G L, in Adobe Photoshop (version CS6 88) and Canvas X (version, 20 Build 390, http://www.canvasgfx.com/). Compare with SI Fig. 1. See text for details.

Analysis of the limb, foot and weight distribution of possible crocodylomorph trackmakers known from Mesozoic body fossils has the potential to shed light on this issue. Study of trackways registered by modern crocodilians during terrestrial walking progression is also informative^21^, and indicates that pes and manus track morphologies are quite similar to those of Mesozoic crocodylomorphs. However, the trackways of well-known extant species such as *Alligator mississippiensis* and *Crocodylus acutus* are generally wider than those reported from the Mesozoic^21,45^ (Fig. 8). Moreover, while it is known that modern species can run bipedally for short distances, trackways produced during such running progression are rare and to the best of our knowledge not recorded or mapped to scales from which morphometric data can be obtained. On the other hand there are videos of modern crocodilian progressing subaqueously with only the pes contacting the substrate in a regular right-left-right cycle^46,47^. Again, the substrate expressions of such subaqueously-registered tracks, corresponding to video footage, have not been recorded. However, such subaqueous progression does potentially provide a plausible mechanism for producing pes only trackways.

While said video footage^46,47^ of modern crocodilians progressing subaqueously shows that they may make tracks with the pes while the manus makes no contact with the substrate, the trackway patterns produced by such ‘punting’ modes of locomotion^48–50^ have not been recorded. We may infer that any punting pes tracks would be incomplete pes toe tracks of the “swim track” variety^49,51^. Therefore, we cannot infer that *B. grandis* ichnosp. nov. tracks and regular trackway patterns represent punting progression.

Modern crocodilian trackways made during normal walking are wide gauge. While similar in overall shape and digit trace proportions, they do not show clear digital pads, but conversely, unlike *B. grandis* ichnosp. nov., they show interdigital web traces. Thus, we can infer that *B. grandis* ichnosp. nov. tracks are different from those of extant crocodilians. This conclusion points again to the likelihood that the *B. grandis* ichnosp. nov. trackmaker was a terrestrial or semi-terrestrial form, with the weight of evidence supporting bipedality^43,44^.

### Distinguishing crocodilian from pterosaurian trackways

An unexpected result of the discovery of *B. grandis* trackway has been to shed light on a the controversial issue of pterosaur locomotion debated since the 1980s^27^ and 1990s^26^: were pterosaurs bipedal or quadrupedal? These debates, mainly concerning relatively small pterosaurian tracks, have largely been resolved in favor of quadrupedalism^52^. However, some uncertainty remained regarding tracks of purported ‘giant’ pterosaurians that were described as ‘enigmatic’ and inferred to have progressed bipedally^28^. These trackways from the Lower Cretaceous, Haman Formation, at the Gain-ri tracksite, Korea were named *Haenamichnus gainensis*^28^ and inferred to represent, large, plantigrade pterodactyloid pterosaurs that might have walked bipedally so that the long wings did not become mired in the substrate. It was further inferred they may have been wading in shallow water.

We can now confirm confidently, that these tracks from the Gain-ri tracksite and others from Adu Island^28^: (SI Fig. 7) are identical to poorly preserved large *Batrachopus* trackways. Thus, they should be removed from *Haenamichnus*^28,50^ and regarded as large poorly preserved batrachopodid tracks. The type specimen then technically becomes *Batrachopus gainensis* (comb nov.). Thus, *H. gainensis* becomes a footnote to ichnotaxonomic history, shown to be an extramorphological expressions large of *Batrachopus*, only recognizable retrospectively after comparison with *B. grandis*. Therefore ichnologists may retrospectively choose to regard *H. gainensis* as a *nomen dubium*, and find little value in the trival name (*gainensis*). Alternatively they may simply refer to the Haman Formation tracks as *Batrachopus* cf. *grandis*.

Note that the Gain-ri and Adu island trackways are from the Haman Formation and so these occurrences indicate a widespread distribution in space (three sites) and time (two formations) of this distinctive apparently bipedal morphotype. The pes tracks from the two Haman Formation sites are also larger (27.5–39.0 cm long), but with trackway proportions (step, stride, pace angulation etc.,) quite similar to those from the Jinju Formation.

The identification of the Haman Formation trackways as poorly preserved large batrachopodid tracks apparently suggests that the trackmakers habitually progressed bipedally. Alternatively the same speculative arguments for apparent rather than real bipedalism would have to be invoked as was the case with the Jinju material. Moreover, in almost all cases the trackways are very narrow gauge with a narrower straddle than seen in modern crocodylians (Fig. 8). It is also of interest that least five sub parallel more or less equally spaced trackways^53^ were registered on the level 4 surface. This suggests either that the trackmakers may have been gregarious, or that they were following a physically controlled route, such as a shoreline, defined by the paleoenvironment^54^.

The overall length of extant crocodylians can be estimated from their tracks using the pes, body length ratio 1:12^55^. Based on this proportion the largest *B. grandis* ichnosp. nov. tracks from the Jinju Formation indicate a trackmaker with length of up to about 3.0 m (pes length 0.25 m × 12) and no less than about 2.16 m (0.18 m × 12). If we include the large poorly preserved tracks from the Haman Formation the overall length of the trackmaker could have been up to 4.68 m (0.39 m × 12).

## Conclusions

The Lower Cretaceous Jinju Formation has yielded the largest known *Batrachopus* track morphotype, and the largest known assemblage, including many well-preserved tracks and trackways with clearly defined digital pad impressions and localized skin traces. These rank high (2–3) on the four point 0-1-2-3 quality of preservation scale^31,32^, and form the basis of the new ichnospecies *Batrachopus grandis* ichnosp. nov.

Surprisingly, the trackways appear to represent bipedal progression which is atypical of all known smaller batrachopodid trackways. This suggests gaits atypical of large crocodylomorphs, except for a few early Mesozoic reports^43,44^. Less well-preserved large batrachopodid tracks from the overlying Haman Formation, previously described as “enigmatic” and incorrectly assigned the pterosaurian ichnogenus *Haenamichnus* (as *H. gainensis*) are reinterpreted as Batrachopodidae tracks, also representing large crocodylomorphs. These apparently also indicate bipedal progression, and increase the database for this trackway morphotype in space and time.

The possibility that *B. grandis* ichnosp. nov. represents quadrupedal progression where manus tracks are not recognized because they registered on a different higher sedimentary surface is not supported by such evidence. Likewise the possibility that ‘unrecognized’ *B. grandis* ichnosp. nov. manus tracks were overprinted by the pes is also not supported by the evidence. Both scenarios, while intriguing and necessary to consider, are based only on inference and negative evidence, rather than the abundant trackway evidence.

The scenario that the trackmakers were progressing by punting through shallow water, only using their hind feet is considered implausible due to the fully plantigrade registration of pes tracks in narrow regular trackways rather that the partial toe-tip tracks that result from punting or swim tracks.

The trackway evidence of a large crocodylomorph with bipedal or facultative bipedal gait in the Lower Cretaceous is surprising, but consistent with terrestrial or semi-terrestrial adaptation, reported from the early Mesozoic trackmakers, and could potentially be supported by future body fossil evidence from the Cretaceous record.

## Supplementary information

Supplementary information.
