# Supplementary material for: Trackway evidence for large bipedal crocodylomorphs from the Cretaceous of Korea
Source: Sci Rep. 2020 Jun 11;10:8680. doi: 10.1038/s41598-020-66008-7 (PMC7289791; doi:10.1038/s41598-020-66008-7)
Supplement: Supplementary file 1 — Supplementary information. [file 41598_2020_66008_MOESM1_ESM.pdf]

Supplementary Information

Trackway evidence for large bipedal crocodylomorphs from the Cretaceous of Korea

Kyung Soo Kim[1], Martin G. Lockley[2], Jong Deock Lim[3], Seul Mi Bae [4], Anthony Romilio[5],

[1] *Department of Science Education, Chinju National University of Education, Shinan-dong, Jinju, Kyungnam, 52673, South Korea*

[2] *Dinosaur Trackers Research Group, University of Colorado Denver, P.O. Box 173364, Denver, CO 80217-3364, USA* *Martin.Lockley@UCDenver.edu* * corresponding author

[3] *Restoration Technology Division, National Research Institute of Cultural Heritage, 132, Munji-ro Yuseong-gu, Daejon, 34122, South Korea*

[4] *Institute of Korea Geoheritage, Chinju National University of Education, Shinan-dong, Jinju, Kyungnam, 52673, South Korea*

[5] *School of Chemistry and Molecular Biosciences, The University of Queensland, Brisbane, Qld 4072, Australia*

This Supplementary Information file deals with four topics related to the main paper. References 1-68 use the same numbering scheme as the main text. References 69-81 are additional.

Contents

1) Material and Methods
2) The distribution of fossil crocodylomorph trackways in space and time
3) Further arguments for and against bipedalism of the *B. grandis* trackmaker.
4) Crocodilian or Pterosaurian: an ongoing debate
5) Supplementary References

1. Material and methods

As discussed in the main text, most trackways attributed to walking crocodylomorphs have been reported as representative of the ichnofamily Batrachopodidae,[2,5] currently known only from the Mesozoic. In order of relative abundance this ichnofamily includes only three ichnogenera: *Batrachopus*[5] abundant in the Lower Jurassic of North America and occurring also in Europe and Africa;[7] *Crocodylopodus*, previously known only from Spain[8] and only recently reported from Korea[12,13] and Iran[45] and *Antipus* known only from a single Jurassic trackway.[10] These ichnotaxa all represent relatively small narrow gauge quadrupeds, inferred to represent

semi-terrestrial or terrestrial forms[3,4] which registered diagnostic trackways from which standard size, shape and trackway configuration measurements are obtained (SI Figs. 1-2). Such batrachopodid trackways represent a different category of ichnological evidence from the “swim tracks” assigned to ichnogenus *Hatcherichnus*, and similar morphotypes that are particularly abundant in the Cretaceous of North America.[24,49] As discussed, here and in the main text the sedimentary facies from which trackways of walking and swimming crocodylomorphs have been reported generally represent different paleoenvironments, which were likely home to different crocodylomorph trackmaker species.[1]

The trackways describe here from the Sacheon Jahye-ri tracksite are unequivocally assigned to the Batrachopodidae (*Batrachopus*) and represent the largest sample of this ichnogenus ever reported from a single site. They also represent the only sample with such large tracks (pes tracks up 24 cm long) (SI Fig. 1) and the only sample to consistently and represent bipeds: but see re-evaluation of trackways from Gain-ri and Adu Island:[28] Fig. 8 in main text.

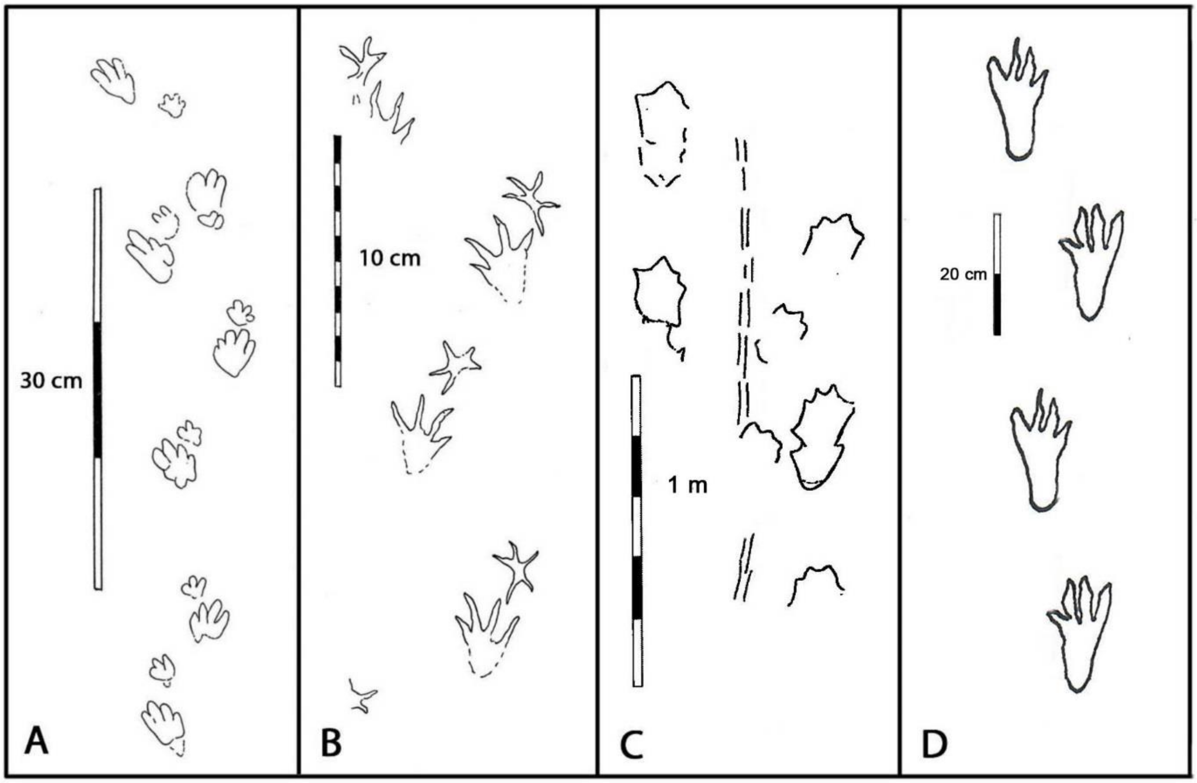

SI Figure 1. A: *Batrachopus* type trackway from Lower Jurassic of New England, B: *Crocodylopodus* form the Lower Cretaceous of Spain,[5] C: *Mehliella* from the Cretaceous of Colorado; D: *Batrachopus grandis* ichnosp. nov. from the Jinju Formation Sacheopn Jahye-ri tracksite, South Korea. Original line drawings made and compiled by M G L, in Adobe photoshop (version CS6 88) and Canvas X (version, 20 Build 390, http://www.canvasgfx.com/). Compare with Fig. 8 (in main text).

The Sacheon Jahye-ri tracksite is a large, gently-southeastward-dipping, exposure, of fine-grained sandstones separated by thin grey mudstones (main text Fig. 2). The outcrop, at the time of the present study had a maximum dimensions of about 20 x 40 m and was created by excavation of bedrock during local construction projects, which required that paleontological surveys be conducted. At least four discrete track-bearing surfaces and one fossil plants-bearing layer were identified, mapped and placed in sequential relationship to one another as levels 1–5 (main text Fig. 2). Due to the necessity of excavating from the top down, track-bearing level 1 is stratigraphically the highest and level 5 the lowest. Collectively the levels have yielded tracks of avian (bird) and non-avian theropods, sauropods, small pterosaurs and the crocodylomorph trackways described here, which are primarily well-preserved on the level 2 and level 4 surfaces. A complete survey of all non-crocodylomorph tracks will require a separate study and publication elsewhere. Almost all crocodylomorph tracks from this site represent pes traces with typical *Batrachopus* morphology. Only two isolated, reasonably complete but small manus tracks have been identified and morphologically attributed to *Crocodylopodus*. They bear no relationship to the large *Batrachopus* trackways either in size or in relation to where they are located in the site.

Many original tracks were collected as successively higher beds were removed to expose underlying track bearing layers. Many of these tracks represent natural casts (convex hyporeliefs) of the crocodylomorph tracks described here, which are the counterparts of the tracks and trackways preserved on the level 2 surface as natural impressions (concave epireliefs). These and other tracks including a few natural impressions are catalogued in the sequence SJ 001 –SJ 140 (SJ = Sacheon Jahye-ri), as part of the mandated rescue and documentation efforts. Most of the tracks were also photographed for archive purposes as well as for the photogrammetric analysis described below.

As detailed in SI Table 1, the sample of tracks recorded here and formally referred to the new ichnotaxon *Batrachopus grandis* ichnosp. nov. includes at least 68 well-preserved individual tracks casts and impressions in the series (SJ 001-140), as well as several in situ trackway segments. The collected casts are notable because at least half of these reveal well-defined, individual digit impressions, in many cases with clearly recognizable phalangeal pad casts separated by inter-pad creases. One preserves skin traces (Fig. 4 main text and SI Fig. 5). Many *Batrachopus* occur in recognizable trackway sequences from which morphometric trackway parameters were obtained. In a minority of cases sharp claw traces and partial skin impression of heel region are preserved. Most of the pes tracks also show a well-defined transverse crease separating the anterior portion of the tracks, with digit traces from the posterior heel region. This transverse crease is often bi-lobed with an anteriorly convex sulcus. In addition to these casts and impressions with well-defined digit and pad traces, ranking as 2-3 on the four point scale (0-1-2-3)[31] there are dozens of additional less well-defined casts and impressions showing the general track outline but not clearly defined digit and pad traces. Specimens for are housed in the Chinju National University of Education.

Representative tracks and trackway segments were also traced on acetate film (Figs. 3, 6 and 8, SI Figs. 1-3) for comparison with the photogrammetric models (Figs. 5,6, SI Fig. 4), and the following standard measurements were obtained: length of track without heel (L), width (W), L/W, length of trace of digits I, II, III and IV, all with heel, with digit III representing total track

length, divarication between all digits (I-II, II-III, and III-IV), plus total divarication (I-IV), step, stride, pace angulation and inner and outer trackway width (SI Table 1, SI Fig. 2).

For photogrammetric purposes we created virtual 3D models of selected portions of the level 2 surface at the Sacheon Jahye-ri tracksite by compiling the photographs collected with a Nikon D600 digital camera (16mm lens). We processed these images using Agisoft Metashape Professional (v.1.5.0) then positioned each virtual model to the centre of the Cartesian coordinate system using Meshlab (64bit_fp v2016.12).[69] We visualized the surface topography using filters in Paraview (version 5.0.0 64 bit);[70] and CloudCompare (v2.6.1 64 bit; www.cloudcompare.org).[71-72]

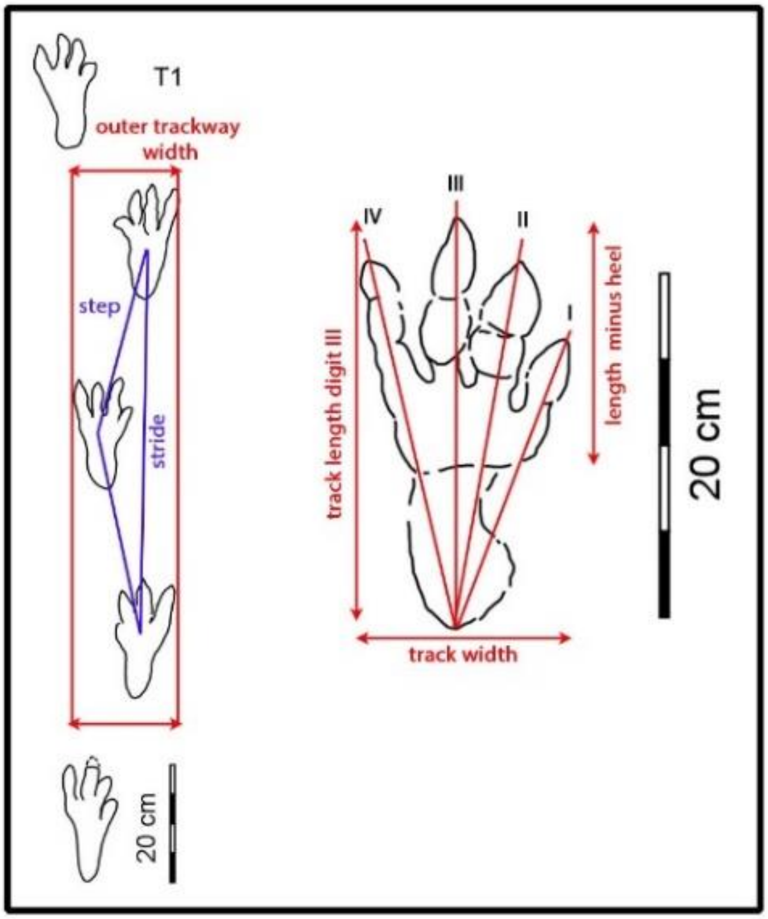

SI Figure 2. Trackway (left) and track (right) parameters measured in the present study. Pace angulation measured as angle between two consecutive steps taken from mid-point of tracks. Inner trackway width measured from lines defining inner margin of track has negative values where tracks overlap trackway mid line. Maximum pes length measured along axis of digit III. Note divarication angle between digits III and IV typically greater than between I and II or II and III. Original line drawings made and compiled by M G L, in Adobe photoshop (version CS6 88) and Canvas X (version, 20 Build 390, http://www.canvasgfx.com/).

SI Table 1 Morphometric parameters for *B. grandis* ichnosp. nov. crocodylomorph trackways and individual tracks from the Sacheon Jahye-ri tracksite. IS = isolated track

Specimen no.	L	W	Length of Pes Digit trace				Divarication Angle of Digit(°)				Step	Stride	PA	Outer TW Width	Inner TW Width	Level	N
			Ⅰ	Ⅱ	Ⅲ	Ⅳ	Ⅰ-Ⅳ	Ⅰ-Ⅱ	Ⅱ-Ⅲ	Ⅲ-Ⅳ							
SJ 013	15.1	8.5	16.0	19.5	23.5	20.7	25.0	7.0	8.0	10.0	–	–	–	–	–	2	1
SJ 037	13.2	10.6	–	–	–	–	–	–	–	–	–	–	–	–	–	2	1
SJ 104	12.7	10.6	–	–	–	–	–	–	–	–	–	–	–	–	–	2	1
SJ 106	11.0	8.7	–	–	20.0	–	30.0	8.0	10.0	12.0	–	–	–	–	–	2	1
SJ 113	11.0	10.5	14.2	17.0	18.8	16.2	37.0	12.0	11.5	13.5	–	–	–	–	–	2	1
SJ 117	11.6	10.2	14.3	18.6	20.7	20.2	29.0	8.0	7.0	14.0	–	–	–	–	–	2	1
SJ 119	12.1	9.5	–	–	–	–	–	–	–	–	–	–	–	–	–	2	1
SJ 121	12.7	9.5	14.0	20.1	22.2	21.6	28.0	5.0	13.0	10.0	–	–	–	–	–	2	1
SJ 128	12.1	9.0	–	–	–	–	–	–	–	–	–	–	–	–	–	2	1
SJ 131	12.0	9.8	14.0	16.9	18.6	17.0	34.0	13.0	7.0	14.0	–	–	–	–	–	2	1
SJ 132	12.1	9.9	15.6	18.5	20.3	19.1	29.0	9.0	8.0	12.0	–	–	–	–	–	2	1
SJ 133	11.4	8.5	12.3	15.9	18.0	17.2	33.0	8.0	12.0	13.0	–	–	–	–	–	2	1
SJ 136	11.1	10.0	–	–	–	–	–	–	–	–	–	–	–	–	–	2	1
SJ 138	14.8	12.5	18.0	22.1	24.0	21.9	33.0	11.0	10.0	12.0	–	–	–	–	–	2	1
SJ 139	12.7	10.2	15.5	19.5	21.5	20.6	28.0	8.0	9.0	11.0	–	–	–	–	–	2	1
a 140	12.2	8.5	13.8	17.4	21.1	19.7	28.0	8.0	8.0	12.0	–	–	–	–	–	2	1
b 140	12.0	–	17.0	21.5	22.5	–	–	8.0	9.0	–	–	–	–	–	–	2	1
IS 1	–	10.5	14.0	17.0	19.1	18.0	38.0	11.0	12.0	15.0	–	–	–	–	–	2	1
IS 4	–	10.7	12.7	18.1	19.3	18.5	37.0	16.0	6.0	15.0	–	–	–	–	–	2	1
IS 5	–	10.0	–	–	18.0	–	–	–	–	–	–	–	–	–	–	2	1
IS 6	–	9.5	–	–	19.0	–	–	–	–	–	–	–	–	–	–	2	1
T 1	11.2	10.4	14.4	18.6	20.3	19.5	32.0	9.6	9.4	12.0	33.9	67.1	155.0	17.0	-2.3	2	5
T 2	–	11.2	15.7	18.4	22.0	21.0	33.0	10.0	9.7	12.3	39.0	74.3	148.0	22.0	0.0	2	3
T 3	–	9.0	–	–	19.0	–	–	–	–	–	34.5	67.0	–	18.0	0.0	2	3
T 4	–	9.5	–	–	20.0	–	–	–	–	–	37.3	71.0	147.0	19.5	2.0	2	3
T 5	–	9.0	–	–	18.0	–	–	–	–	–	31.5	50.8	135.0	20.0	3.0	2	3
T 6	–	10.0	–	–	22.0	–	–	–	–	–	45.0	–	–	–	–	2	2
T 7	–	8.5	–	–	21.0	–	–	–	–	–	36.0	69.0	152.0	18.0	0.0	2	3
T 8	–	8.0	–	–	20.0	–	–	–	–	–	34.0	64.5	140.0	19.0	1.0	2	3
T 9	–	10.0	–	–	22.0	–	–	–	–	–	47.0	–	–	–	–	2	2
T 10	–	9.5	–	–	21.0	–	–	–	–	–	36.0	–	–	–	–	2	2
T 11	–	8.0	–	–	20.0	–	–	–	–	–	39.0	74.0	140.0	22.0	5.5	4	3
T 12	–	9.5	–	–	22.0	–	–	–	–	–	35.5	66.5	140.0	21.0	4.0	4	3
T 13	–	9.0	–	–	25.0	–	–	–	–	–	40.0	74.0	152.0	17.0	0.0	4	3
T 14	–	10.0	–	–	21.5	–	–	–	–	–	36.5	72.5	160.0	17.5	-2.5	4	3
T 15	–	9.0	–	–	20.0	–	–	–	–	–	42.0	81.0	165.0	13.0	-4.0	4	3
T 16	-	9.2	-	-	21.8	-	25.7	7.4	8.8	-	34.3	66.3	149.0	17.9	0.0	2	4
T 17	-	9.6	-	-	21.8	-	27.9	7.8	9.6	-	27.3	48.4	119.0	25.7	6.3	2	5
**Average**	**12.3**	**9.6**	**14.8**	**18.6**	**20.7**	**19.4**	**31.0**	**9.3**	**9.3**	**12.5**	**37.0**	**67.6**	**146.3**	**19.1**	**0.9**	Total	**74**

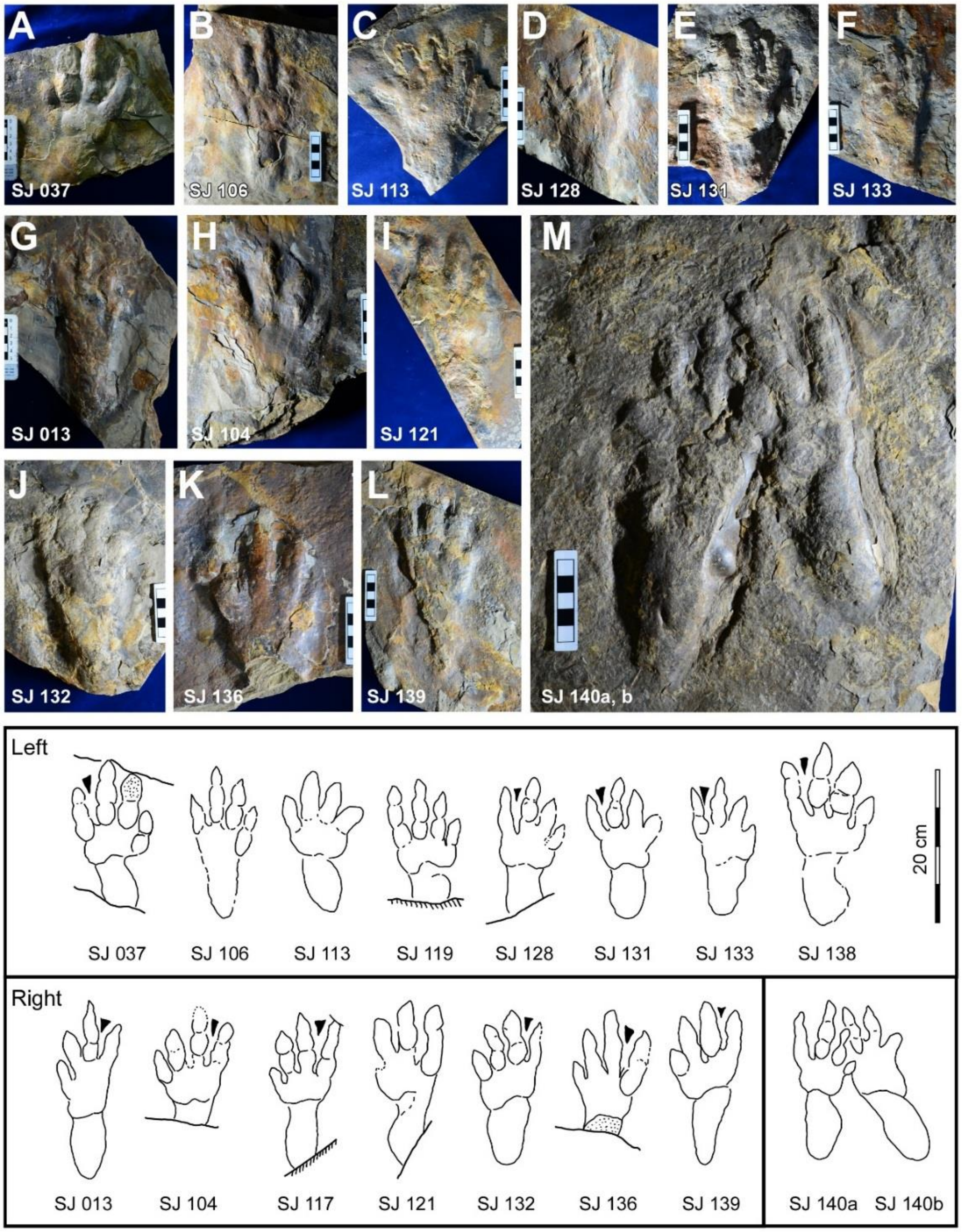

SI Figure 3. Photographs and outline drawings of *Batrachopus grandis* ichnosp. nov. track casts. Black triangle indicates that divarication between digits III and IV is greater than between other digits. Photographs and line drawings made and compiled by K-S K and M G L in Adobe photoshop (version CS6 88) and Canvas X (version, 20 Build 390, http://www.canvasgfx.com/). Compare with Figs. 3-6 (main text) and SI Fig. 4.

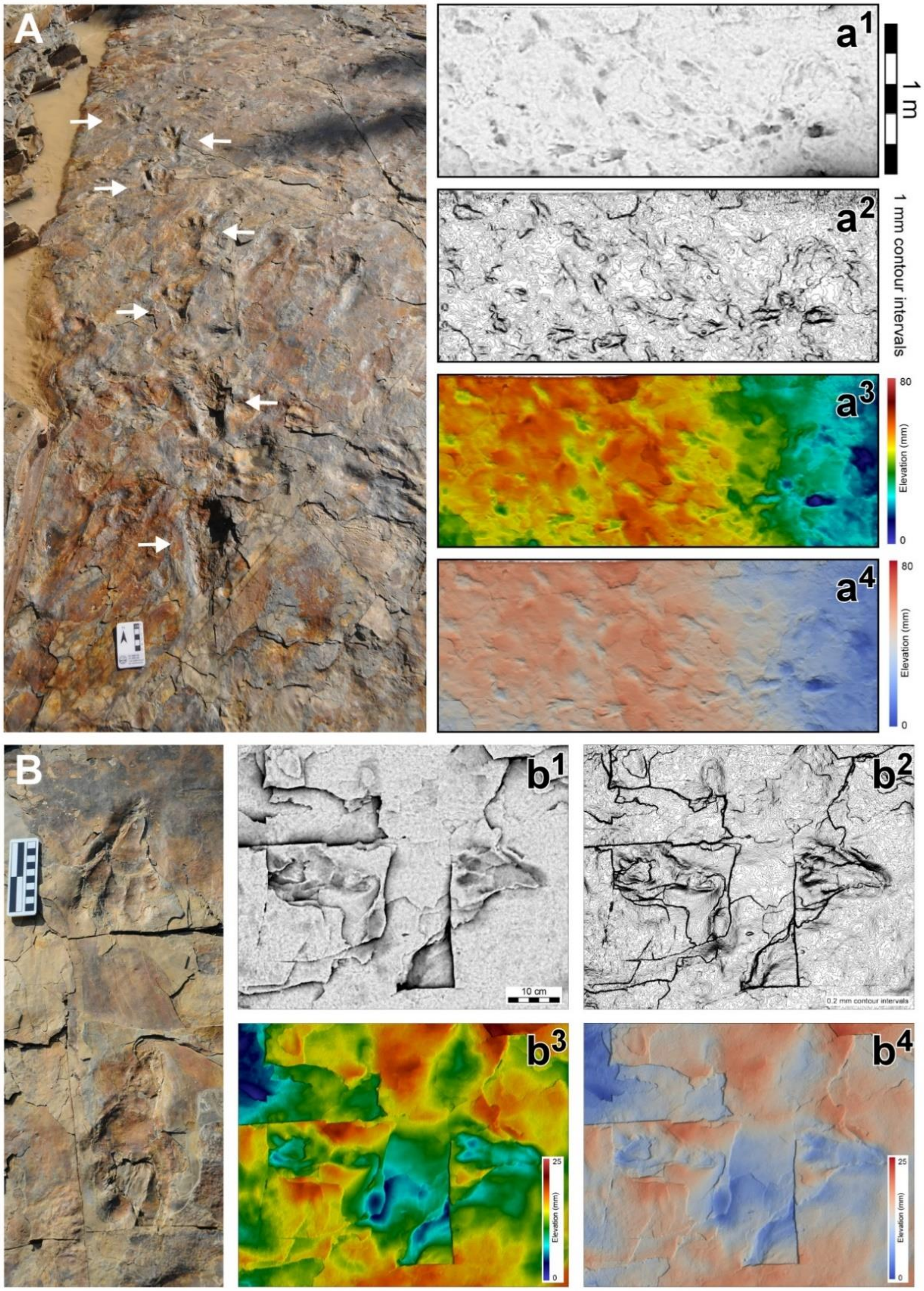

SI Figure 4. Photographs and 3D images of trackway segments. A-B: Photos of trackway 1 and 2 respectively; a-b: 3D images of trackway 1 and 2. Photos and 3D images made and compiled by K-S K and A R, in Adobe photoshop (version CS6 88) and Canvas X (version, 20 Build 390, http://www.canvasgfx.com/). Compare with Figs. 3-6 (main text) and SI Fig. 3.

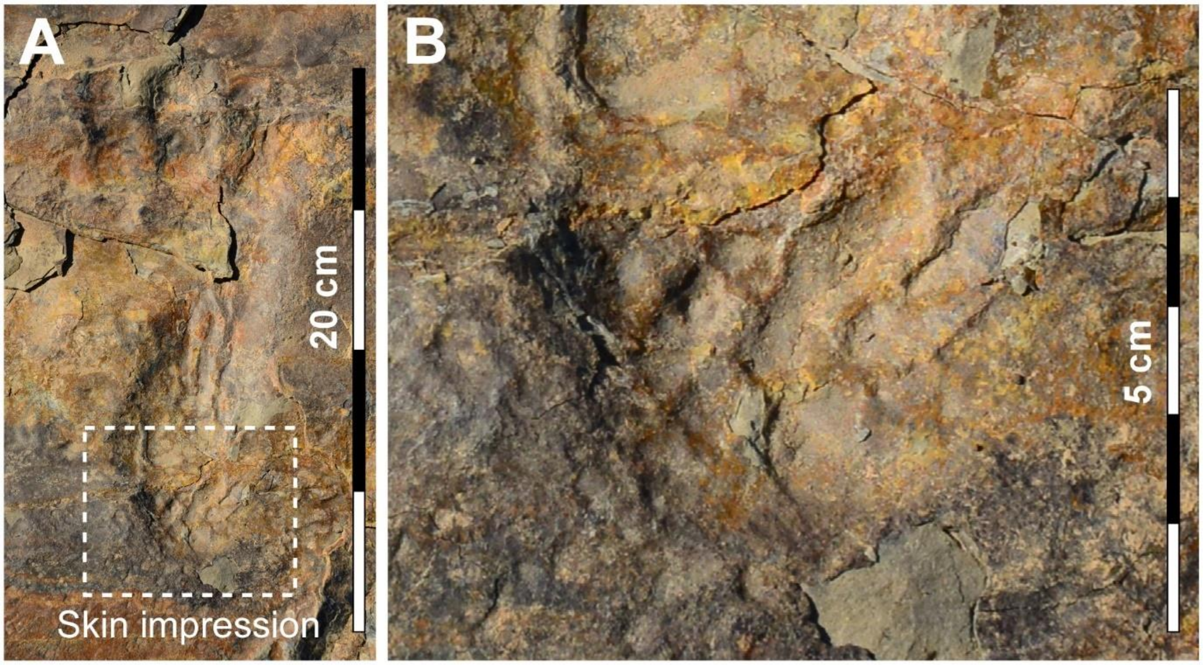

SI Figure 5. Detail of *B. grandis* ichnosp. nov. pes track with skin scale traces in posterior region of heel. Note that polygonal scale traces are large (~ 1.0 cm in diameter). Photograph by J-W Kang and modified by K-S K in Canvas X (version, 20 Build 390, http://www.canvasgfx.com/). Compare with Fig. 4 main text.

2. The distribution of fossil crocodylomorph trackways in space and time

Although the well-known crocodylomorph trackway *Batrachopus* is abundant in the Lower Jurassic throughout North America[3,47] and also reported from the Lower Jurassic of Europe [6] and South Africa, [7] it has only once been reported from the Cretaceous, at a single site in Thailand, in an occurrence described as surprising,[29] even though the region has produced body fossils of dwarf, normal-sized and even giant crocodiles. Additional reports of small crocodylomorph tracks from this Lower Cretaceous of this area[73] do not describe, illustrate or name the morphotype(s) in any detail. Nevertheless it should be stressed that reports of such large batrachopodid morphotypes, as reported here from in Korea, have never previously been reported anywhere in the global track record.

However, the track rich Jinju Formation has recently yielded a large number of *Crocodylopodus* trackways[12,13] which represent the first unequivocal assemblages, with diagnostic trackways known from the Korean Cretaceous (SI Fig. 6) and only the second report, of trckways after those recorded from the *Crocodylopodus* type area in Spain.[8] *Batrachopus* and *Crocodylopodus* both belong in the same ichnofamily (Batrachopodidae)[5] but differ in detail, including the relatively large size of the manus in *Crocodylopodus*, and the slenderness of the digit traces in both manus and pes.

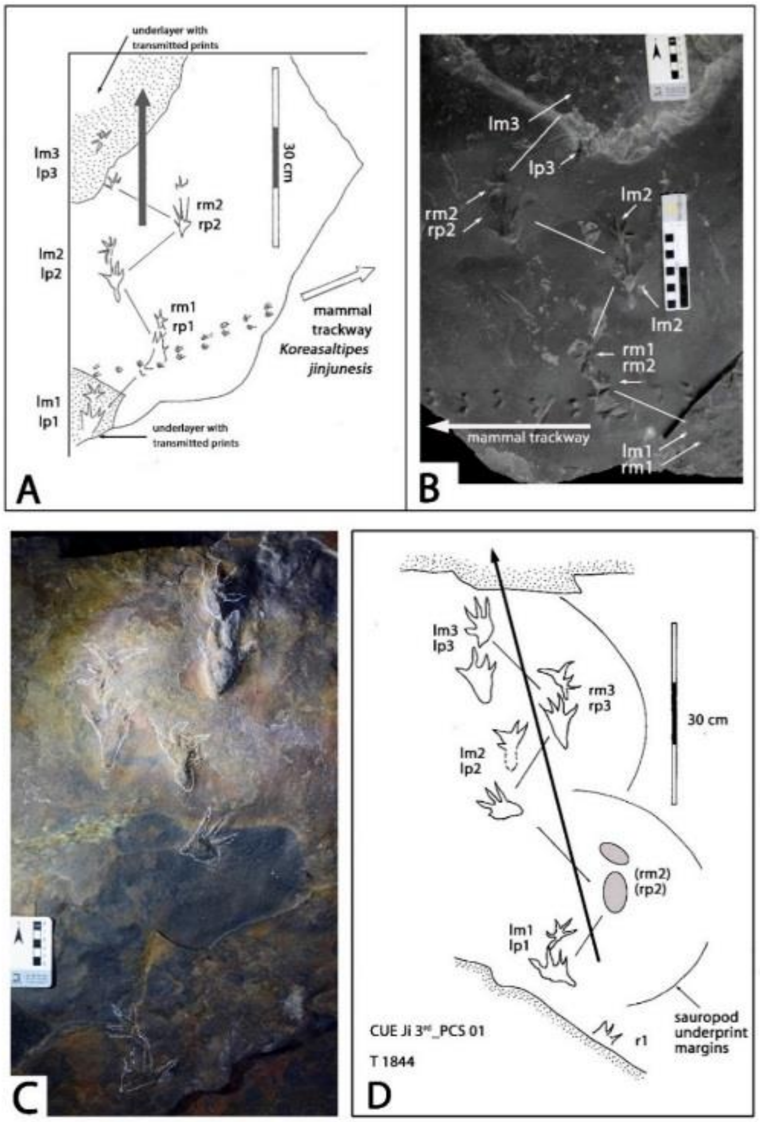

SI Figure 6. Line Drawing A and photograph B of *Crocodylopodus* trackway (CUE E4 C001) in the Jinju Pterosaur Tracks Museum. Photograph C and line drawing D of *Crocodylopodus* trackway (CUE Ji 3rd _PCS 01) in the Jinju Pterosaur Tracks Museum. Photographs and line drawings by K-S K and M G L in Adobe photoshop (version CS6 88) and Canvas X (version, 20 Build 390, http://www.canvasgfx.com/).

Although at least 100 Cretaceous tetrapod tracksites are known from China, none have yielded unequivocal crocodylomorph tracks and potential body fossils are also rare or absent.[1] A possible Jurassic report from China labelled as ichnogenus *Kuangyuanpus*, remains enigmatic[67] while another from the Cretaceous[67] is has been reinterpreted as of turtle affinity.[74,75] This lack of crocodylomorph track evidence from China,[1] at the time also lacking from Korea, was inferred to indicate a lack of suitable habitat for aquatic crocodilians which were abundant in the Cretaceous of North America where their swim tracks, mostly ichnogenus *Hatcherichnus*, are known from dozens of localities.[49] However, given that almost all *Batrachopus* and

Crocodylopodus trackways from both the Jurassic and Cretaceous, including the large *Batrachopus* described here, represent narrow gauge trackmakers progressing over presumed emergent (terrestrial), not subaqueous, substrates, it is necessary to consider the likely differences in distribution and significance of track assemblages attributed to aquatic (swimming or punting) crocodylians and those with more semi-terrestrial or terrestrial adaptations. A rare Asian exception of crocodilian swim traces was recently reported from the Upper Cretaceous of Mongolia and represents a small trackmaker (mean pes footprint length, 7.7 cm).[50]

Unlike the trackways of *Batrachopus* and *Crocodylopodus* which indicate walking progression, trackways of *Hatcherichnus*[24] which are common in North America, especially in the mid Cretaceous Dakota Group represent swim tracks.[49] In fact the ichnogenus has been used to label the *Hatcherichnus* ichnofacies,[77] representative of the *Charachichnos* swim track ichnofacies. Type *Hatcherichnus* reveals incomplete manus and pes tracks associated with both sinuous and sublinear tail traces which help define the trackway midline. Compare with the belly and tail drag traces reported for wide straddle (trackway width) in the "low walk" trackways of *Mehliella*[25] and the Mongolian trackmaker.[50] Individual pes and manus swim tracks are typically incomplete, except in rare cases, often consisting of distal toe tip traces, as has been known for Dakota Group tracks since the 1980s.[51] Other named crocodylomorph tracks explicitly attributed to swimming crocodilians include *Anticusuchipes*[48] from the Eocene of Washington state, USA. This latter report notes some of the differences between the high and low walking styles of modern crocodilians[21] and the 'punting' mode of "submerged locomotion" which leads to irregular and intermittent contact of the trackmaker with subaqueous substrates.[48,57] It is beyond the scope of this paper to discuss the variation in crocodilian swim tracks from the Mesozoic and Cenozoic, except to note that as swim tracks are inherently incomplete representations of the trackway patterns made by their trackmakers when registering walking trackways on land. Likewise wide straddle low walk or semi-sliding trackways[50] also raise challenging ichnotaxonomic questions. For example, are swim track ichnotaxa inherently incomplete expressions of diagnostic trackmaker morphology, and therefore suspect? Likewise, are such 'incompletely registered' traces, like the ichnotaxa *Anticusuchipes* and *Borealosuchipus*[58] subjective junior synonyms of earlier named forms like *Hatcherichnus*? (Table 1, main text). Such questions are important to address the potential problem of proliferation of ichnotaxonomic names for incomplete tracks and trackway segments that may on close scrutiny be *nomina dubia*: i.e., not diagnostic, or diagnostically distinguishable from other ichnotaxa. These questions are also relevant here if the *B. grandis* ichnosp. nov. trackway is interpreted as incomplete (without manus traces) due to subaqueous progression. However, this concern does not rise substantive ichnotaxonomic issues if the trackways truly represent bipeds.

3. Further arguments for and against bipedalism of the *B. grandis* trackmaker

It is evident that type *Batrachopus*, and *B. grandis* ichnosp. nov. described here typically show well-defined and relatively broad digit traces including pad impression, whereas *Crocodylopodus* has slender tapering digit traces reminiscent of penetrative tracks, characteristic of tracks made in certain soft fine-grained, well-laminated sediment:[20] SI Fig. 1. It is necessary to consider if such sub-optimal modes of preservation might lead to viewing *Crocodylopodus* as an

extramorphological as a poorly preserved (extramorphological) expression of crocodylomorph tracks that otherwise resemble *Batrachopus*? Thus it is inferred that the two ichnogenera should be considered members of the same ichnofamily (Batrachopodidae).[5] However, they noted that there are differences, in detail, as outlined in the descriptions[3,4] for *Batrachopus* and *Crocodylopodus*.[8] For example, while we know the phalangeal pad formula for *Batrachopus* and recognize that digit III is the longest, neither of these details can be confirmed in morphometric detail for *Crocodylopodus*. However *Crocodylopodus* exhibits less outward pes rotation than quadrupedal *Batrachopus,* greater digit divarication, and a smaller heel, with what was referred to as a "little heel" trace. Moreover, the evidence that throughout all samples, the *B. grandis* ichnosp. nov. trackmaker was bipedal while the author of *Crocodylopodus* was not, is further evidence of differences not easily attributable to the vagaries of preservation.

Admittedly, the evidence that *B. grandis* ichnosp. nov. appears to have been bipedal is potentially puzzling, given that no bipedal crocodylomorphs have yet been reported from the Cretaceous. However, such large trackmakers are known from the early Mesozoic [24.44] and we also now know that *Batrachopus*, representing semi-terrestrial Early Jurassic crocodylomorph trackmaker [3,4] is also known from the Cretaceous of Asia.[29] as reviewed in the main text no compelling sedimentological (preservational), ichnological or original locomotor evidence can be found to refute the interpretation. Nevertheless it is possible to argue, in theory, that the manus tracks were in all cases obscured by overprinting by the pes. However, in order to make this case one must argue for ipsolateral rather than the contralateral motion of the limbs typical of extant crocodylians. Generally complete primary overlap, where the pes overprints the manus track registered in ahead of it on the same side is impossible with contralateral locomotion, even if slight overlap of the anterior pes trace on the posterior of the manus trace is possible. The only other ichnologically credible explanation for overprinting would be secondary or tertiary overlap, as described for some long bodied forms[78,79] but such locomotor dynamics have not been reported for crocodylians.

As mentioned in the main text and touched on in the literature,[35,48.50] it is know that extant crocodylians sometimes "punt" with their hind feet while moving subaqueously, without registering front footprints. However video footage [46,47] indicates that they do not register fully plantigrade pes impressions, rather they register incomplete and irregularly distributed swim tracks of the type well-known from the track record.[51] Likewise there is no evidence that this mode of progression could possibly create regular narrow gauge trackways with the configuration seen repeatedly in *B. grandis* ichnosp. nov. trackways.

Another argument that would potentially refute the case for crocodilian bipedalism here inferred for the *B. grandis* ichnosp. nov. trackmaker would be to postulate a non-crocodylian trackmaker. Such a potential argument would require identifying a trackmaker with the right pes morphology. Theropods which are also archosaurs, like crocodylains, have digital pad formulas of 2-3-4 for digit II, III and IV respectively, but the plantigrade digit I and heel configurations of *Batrachopus* are quite different. Theropods also have a tridactyl not a pentadactyl manus. So a theropodan interpretation does not cast obvious doubt on the inference that batrachopodid trackways have crocodylomorph affinities. As noted below, and in the main text, trackways much like poorly-preserved *B. grandis* ichnosp. nov. from two tracksites in Haman Formation were initially named *Haenamichnus gainensis* (now *B. gainensis* comb. nov.) and attributed to bipedal pterosaurs. However, we here show that this interpretation is incorrect for at least two compelling reasons: a) pterosaurian trackways indicate quadrupedalism (see following section),

and b) pterosaurian trackways are wide gauge (Fig. 8). Given that we now identify three sites, and four stratigraphic levels, with multiple *B. grandis* ichnosp. nov. and *B. grandis*-like trackways, none of which show a single associated manus track, it strains credulity to argue against a bipedal trackmaker in the face of such abundant and consistent evidence.

4. Crocodilian or Pterosaurian: an ongoing debate

The potential to confuse crocodilian and pterosaurian tracks arose in the1980s when the distinctive trackway of a quadruped described as *Pteraichnus* from the Late Jurassic of Arizona,[80] was reinterpreted as pterosaurian.[27] This interpretation was refuted[26,52] by compelling evidence that *Pteraichnus* was indeed pterosaurian, quadrupedal and widely distributed in the North American Jurassic, as well as at various Cretaceous sites.[26,56] This refuted arguments based on claim that pterosaurians walked bipedally,[81] even though it is well-known that extant crocodilians progressed quadrupedally.[21]

Studies of the trackways of the Late Cretaceous ichnospecies *Haenamichnus uhangriensis,*[56] the first pterosaurian trackway reported from Asia, correctly inferred a large pterosaurian trackmaker, based on the presence of diagnostic manus tracks, and a pterosaurian trackway pattern (Fig. 8). However, the registration of the pes in *H. uhangriensis* is sub-optimal, not showing discretely impressed digit traces. The subsequent discovery of other large, elongate pes only trackways at two sites (Gain-ri and Adu Island) in the Lower Cretaceous Haman Formation, were described as "Enigmatic giant pterosaur tracks, named *Haenamichnus gainensis,*[28] and attributed to a bipedal trackmaker possible wading in shallow water. The discovery of the *Batrachopus* tracks from Sacheon Jahye-ri therefore requires re-examination of the inference that *H. gainensis* was a bipedal pterosaurian, or indeed whether pterosaurs ever progressed bipedally. As discussed in the main text we infer that *H. gainensis* represents a crocodilian, allowing the trivial name to be transferred to *Batrachopus* as "*B. gainensis*" *comb. nov*., as a historical footnote. Some ichnologists might simply dismiss *H. gainensis* as a *nomen dubium*.

The difference in footprint length between the Jinju Formation (?Aptian) tracks (FL 18.0-24.0 cm) and those from the younger (?Albian) Haman Formation (27.5–39.0 cm) suggest that the younger trackmakers were larger. However the poor quality of preservation of the Haman tracks might mean that their size was overestimated. As part of this study we reexamined the "*gainensis*" trackways from the Gain-ri and Adu Island[28] (SI Fig. 7) and can definitively state that these are mostly identical to poorly preserved large *Batrachopus* trackways except for size differences: i.e., they represent narrow gauge ostensibly bipedal trackmakers. Thus, they should be removed from ichnogenus *Haenamichnus* and no longer be attributed to pterosaurs.

So surprising as it may be to find trackway evidence strongly suggestive of large bipedal crocodylomorphs in the Cretaceous, the suggestion is not unprecedented for the clade as a whole. In fact, the Cretaceous trackmakers may have been descendants of, or forms convergent with, bipedal, early Mesozoic ancestors.[7,44] On the other hand, the formerly-reported enigmatic tracks interpreted as "surprising" or anomalous evidence of large bipedal pterosaurs[28] can now be dismissed as having been based on a case of mistaken identity.

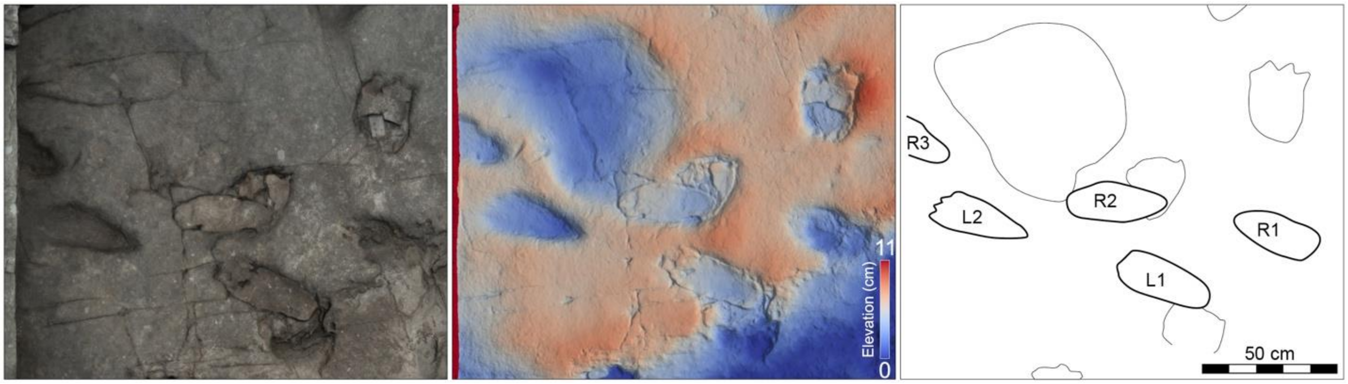

SI Figure 7. 3D image of large biped narrow gauge trackway (cf. *Batrachopus grandis* ichnosp. nov.) consisting of five tracks R1-L1-R2-L2-R3 from the Haman Formation (Adu Island) previously misinterpreted as the trackway of a large bipedal pterosaur (*H. gainensis*). See text for details. Compare with Fig. 8 and SI Fig. 1.

Supplementary references.

References 69-81 are supplemental to references 1-68 in main text

69) Cignoni, P., *et al*. MeshLab: An Open-Source Mesh Processing Tool. *Sixth Eurographics Italian Chapter Conference* (2008)

70) Ahrens, J., Geveci. B. & Law, C. ParaView: An End-User Tool for Large Data Visualization. *Visualization Handbook,* (Elsevier, 2005).

71) Romilio, A., *et al*. A multidisciplinary approach to digital mapping of dinosaurian tracksites in the Lower Cretaceous (Valanginian-Barremian) Broome Sandstone of the Dampier Peninsula, Western Australia. *PeerJ* **5**:e3013; DOI 10.7717/peerj.3013 (2017).

72) Xing, L., *et al*. Multiple parallel deinonychosaurian trackways from a diverse dinosaur track assemblage of the Lower Cretaceous Dasheng Group of Shandong Province, China. *Cretaceous Research* **90**, 40-55 (2018).

73) Kozu, A., *et al*. Dinosaur footprint assemblage from the Lower Cretaceous Khok Kruat Formation, Khorat Group, northeastern Thailand. *Geoscience Frontiers* **8**, 1479-1493 (2017).

74) Lockley, M. G., Li, R., Matsukawa, M. & Li, J. Tracking Chinese crocodilians: *Kuangyuanpus*, *Laiyangpus* and implications for naming crocodylian and crocodylian-like tracks and associated ichnofacies. *New Mexico Museum of Natural History and Science Bulletin* **51**, 99-108 (2010).

75) Lockley, M. G., Xing, L. & Xu, X. The "lost" holotype of *Laiyangpus liui* Young (1960) (Cretaceous, Shandong Province, China) is found: implications for trackmaker identification, ichnotaxonomy and interpretation of turtle tracks. *Cretaceous Research* **95**, 260-267 (2019).

76) Lockley, M. G., A large assemblage of dinosaur tracks from a valley fill deposit in the Dakota Sandstone, central Utah: the Cetaceous fossil footprint database expands westward**.** *New Mexico Museum of Natural History and Science Bulletin* **79**, 375-385 (2018)

77) Hunt, A. P. & Lucas, S. G. Tetrapod ichnofacies: A new paradigm. *Ichnos* **14**, 59-68 (2007).

78) Haubold, H. Ichnia Amphibiorum et Reptilorium fossilium in *Handbuch der Paläoherpetologie*, *Tiel 18* (ed. O. Kuhn) 1-124 (Gustav Fischer Verlag, Stuttgart, 1971).

79) Haubold, H. Saurierfährten. 231 p. (A Ziemsen Verlag, 1984).

80) Stokes, W. A. S. Pterodactyl tracks from the Morrison Formation. *Journal of Paleontology* **31**, 952-954 (1957).

81) Padian, K. A functional analysis of flying and walking in pterosaurs. *Paleobiology* **9**, 218-239 (1983).
